# The Benefits of Whole Genome Sequencing for Foodborne Outbreak Investigation from the Perspective of a National Reference Laboratory in a Smaller Country

**DOI:** 10.3390/foods9081030

**Published:** 2020-08-01

**Authors:** Stéphanie Nouws, Bert Bogaerts, Bavo Verhaegen, Sarah Denayer, Florence Crombé, Klara De Rauw, Denis Piérard, Kathleen Marchal, Kevin Vanneste, Nancy H. C. Roosens, Sigrid C. J. De Keersmaecker

**Affiliations:** 1 Department of Expertise and service provision, Transversal activities in Applied Genomics, Sciensano, 1050 Brussels, Belgium; Stephanie.Nouws@Sciensano.be (S.N.); Bert.Bogaerts@Sciensano.be (B.B.); Kevin.Vanneste@Sciensano.be (K.V.); Nancy.Roosens@Sciensano.be (N.H.C.R.); 2 Department of Information Technology, IDLab, imec, Ghent University, 9052 Ghent, Belgium; Kathleen.Marchal@ugent.be; 3 National Reference Laboratory for Shiga Toxin-Producing *Escherichia coli* (NRL-STEC), National Reference Laboratory for Foodborne Outbreaks (NRL-FBO), Department of Infectious diseases in humans, Foodborne Pathogens, Sciensano, 1050 Brussels, Belgium; Bavo.Verhaegen@Sciensano.be (B.V.); Sarah.Denayer@Sciensano.be (S.D.); 4 Department of Microbiology and Infection Control, National Reference Center for Shiga Toxin-Producing *Escherichia coli* (NRC-STEC), Vrije Universiteit Brussel (VUB), Universitair Ziekenhuis Brussel (UZ Brussel), 1090 Brussels, Belgium; Florence.Crombe@uzbrussel.be (F.C.); Klara.DeRauw@biomerieux.com (K.D.R.); Denis.Pierard@uzbrussel.be (D.P.); 5 Department of Plant Biotechnology and Bioinformatics, Ghent University, 9052 Ghent, Belgium; 6 Department of Genetics, University of Pretoria, Pretoria 0083, South Africa

**Keywords:** whole genome sequencing, WGS, foodborne outbreak investigation, Shiga toxin-producing *Escherichia coli*, STEC, food safety, surveillance

## Abstract

Gradually, conventional methods for foodborne pathogen typing are replaced by whole genome sequencing (WGS). Despite studies describing the overall benefits, National Reference Laboratories of smaller countries often show slower uptake of WGS, mainly because of significant investments required to generate and analyze data of a limited amount of samples. To facilitate this process and incite policy makers to support its implementation, a Shiga toxin-producing *Escherichia coli* (STEC) O157:H7 (*stx1*+, *stx2*+, *eae*+) outbreak (2012) and a STEC O157:H7 (*stx2*+, *eae*+) outbreak (2013) were retrospectively analyzed using WGS and compared with their conventional investigations. The corresponding results were obtained, with WGS delivering even more information, e.g., on virulence and antimicrobial resistance genotypes. Besides a universal, all-in-one workflow with less hands-on-time (five versus seven actual working days for WGS versus conventional), WGS-based cgMLST-typing demonstrated increased resolution. This enabled an accurate cluster definition, which remained unsolved for the 2013 outbreak, partly due to scarce epidemiological linking with the suspect source. Moreover, it allowed detecting two and one earlier circulating STEC O157:H7 (*stx1*+, *stx2*+, *eae*+) and STEC O157:H7 (*stx2*+, *eae*+) strains as closely related to the 2012 and 2013 outbreaks, respectively, which might have further directed epidemiological investigation initially. Although some bottlenecks concerning centralized data-sharing, sampling strategies, and perceived costs should be considered, we delivered a proof-of-concept that even in smaller countries, WGS offers benefits for outbreak investigation, if a sufficient budget is available to ensure its implementation in surveillance. Indeed, applying a database with background isolates is critical in interpreting isolate relationships to outbreaks, and leveraging the true benefit of WGS in outbreak investigation and/or prevention.

## 1. Introduction

Safety of the food chain is assured, amongst others, by regular sampling of a broad diversity of food matrices. Through the elimination of identified, contaminated food matrices, these monitoring systems aid in preventing foodborne outbreaks. When the detection of pathogens with similar characteristics in the human population is exceeding expected numbers [1] within a limited time period or geographical area, an outbreak is suspected. In this case, the goal lies in its rapid confinement by tracing and eliminating the causal food source. This is based on epidemiological interrogation of cases, sampling of suspect food matrices, and matching strains isolated from patients with those collected from suspect food. Conventional methods for pathogen detection and characterization are species-specific and consist of: (i) phenotypic tests, such as sequential selective culturing steps and antimicrobial resistance (AMR) susceptibility tests; (ii) biochemical assays, such as agglutination tests for, e.g., serotype determinations; and (iii) genotypic approaches, such as (quantitative) polymerase chain reaction ((q)PCR) for specific gene detection [2,3]. During foodborne outbreak investigations, identification of relationships between pathogenic strains is, dependent on the species, conventionally performed by pulsed-field gel electrophoresis (PFGE), multilocus variable number tandem repeats analysis (MLVA), multilocus sequence typing (MLST), and insertion sequence (IS) typing [3,4,5,6,7]. In multiple countries worldwide [4,8,9,10,11], these conventional methods are systematically being replaced by whole genome sequencing (WGS), providing information for serotyping, virulence and AMR genotyping through a single approach. Moreover, by virtue of its single nucleotide resolution, the ultimate level of discriminatory power is acquired to investigate phylogenetic relationships between isolates, as part of foodborne outbreak investigations.

Despite numerous studies describing the added value of WGS over conventional methods in foodborne outbreak investigation [4,8,9,10,11], introduction in routine surveillance/monitoring applications remains difficult, mainly in smaller and/or less developed countries [12,13,14]. Although WGS application is often encouraged by their competent authorities, only limited budgets are made available by their respective policy makers, which hinders the building of WGS capacity (i.e., for bioinformatics knowledge, NGS instrument investment, mature sampling strategies, training of laboratory personnel, data storage, generation of data for all isolates, etc.). Moreover, in smaller countries, the number of currently collected isolates can be limited, creating a vicious circle on the false perspective that investment in WGS is redundant, compared to using conventional methods. This lack in resources to support WGS capacity building is visible in public health sectors (i.e., National Reference Centers, NRC; for clinical isolates), but especially in food safety practices (i.e., National Reference Laboratories, NRL; for food isolates) [12,13]. However, using the same methodology for both human and food isolates is crucial to allow sound outbreak investigation. Ideally, this approach should be globally harmonized with regard to the increased risk for multi-national outbreaks, partly because of international food trading [15]. To facilitate the process of full WGS implementation both in public health as well as in food safety practices, it is therefore crucial that national policy makers are convinced of the benefits of WGS, even in smaller countries, to give their financial support [15]. For example, in Belgium, financial resources for WGS of food isolates are solely available for a very limited amount of isolates in the scope of large outbreak investigations, and if human isolates are available. With the aim of providing evidence-based arguments for its added value to the national policy makers, we retrospectively analyzed two Belgian outbreaks using WGS, and compared the results to those initially obtained with conventional methods for (i) isolate characterization, and (ii) the phylogenetic study of isolate relatedness. Shiga toxin-producing *Escherichia coli* (STEC) was chosen as case study because of earlier recommendations to apply molecular techniques that enable the detection of its wide range of virulence genes, and increasing the discriminatory power to differentiate unrelated isolates. Indeed, this is indispensable for STEC because of its rapid evolution [16,17,18]. The two largest STEC outbreaks in Belgium over the past 10 years were selected [19,20], for which conventional methods in combination with available epidemiological data were and were not capable of initially resolving the outbreaks, respectively. The observed added value was subsequently put in the perspective of national and international challenges that are currently being addressed.

## 2. Materials and Methods

### 2.1. Conventional Methods Applied for the Initial Outbreak Investigation

**Figure 1:**
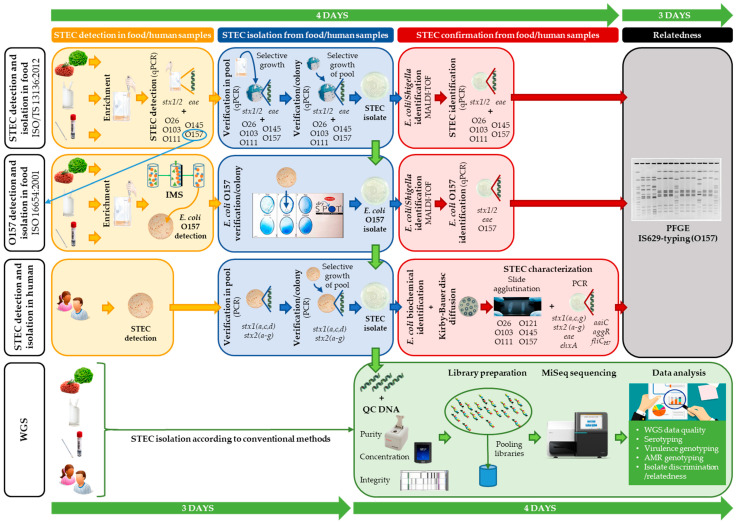
A schematic overview comparing the conventional methods and whole genome sequencing (WGS) workflow as applied in outbreak investigation. The routinely applied conventional methods for food samples (according to ISO/TS 13136:2012 (for detection and isolation of Shiga toxin-producing *Escherichia coli* (STEC)) and ISO 16654:2001 (for detection and isolation of *Escherichia coli* O157)) and for human samples are depicted. Shortly, according to ISO/TS 13136:2012, screening for STEC is performed through qPCR detection of *stx1*, *stx2,* and *eae* in enriched food or swab samples. In case of *stx* and *eae* detection, serogroup (i.e., O-type) determination is also performed with qPCR. STEC isolation is attempted through selective growth on CHROMagar STEC. Both qPCR assays are repeated on a sweep of colonies (pool) for STEC detection, and subsequently on separate colonies to enable STEC isolation. Identification of *E. coli/Shigella* is performed with MALDI-TOF mass spectrometry (MS), and the qPCRs are repeated for STEC identification and confirmation. When a STEC O157 is detected with the ISO/TS 13136:2012 workflow (through qPCR; depicted with a blue arrow), isolation can also be attempted through the serogroup specific workflow (ISO 16654:2001), as was performed for the food isolates of the outbreaks analyzed in this study. Shortly, according to ISO 16654, *E. coli* O157 in enriched food or swab samples are captured using immunomagnetic beads (i.e., immunomagnetic separation (IMS)), and selectively grown on a CT-SMAC plate for *E. coli* O157 detection. Through the Oxoid-Latex test, *E. coli* O157 identification is performed on single colonies, to enable their isolation. The isolated *E. coli* O157 is then confirmed for *E. coli* through MALDI-TOF MS, and for STEC O157 through PCR detection of *stx1, stx2, eae,* and O157 (e.g., *rfbE*). Human samples are selectively grown on (CT)-SMAC biplates for STEC detection. A sweep of colonies (pool) is tested for *stx* presence (including subtyping). STEC is verified through PCR on single colonies grown ((CT)-SMAC) from the PCR positive pool. Isolated STEC is then confirmed as *E. coli* through biochemical identification tests (citrate, mobility indole urease, indole, urease, β-glucuronidase, and sorbitol). Moreover, all human isolates are phenotypically tested for antimicrobial resistance (AMR) through disc diffusion. STEC characterization is further performed through PCR detection of multiple virulence genes, and serogroup determination through slide agglutination. IS629-typing and pulsed-field gel electrophoresis (PFGE) are performed to investigate the relationship between human and food isolates. Completion of these conventional workflows takes approximately 7 working days (indicated by the green time line). With WGS, STEC isolation (according to the conventional methods) is followed by DNA extraction, DNA quality control, and subsequent Nextera XT library preparation for MiSeq sequencing. From the obtained data, all isolate characteristics, and the relatedness between the isolates are retrieved with one approach, with a higher resolution (approximately 7 days (i.e., five working days as two days are used for sequencing), indicated by the green time line). More details concerning the applied workflows are available in the Materials and Methods section and the Supplementary Methods.

The initial investigation was performed with conventional methods (Figure 1) during the respective outbreak courses (for the Limburg 2012 outbreak described in Braeye et al. [6]).

Detection and isolation of O157:H7 isolates in food and carcass swab samples was performed at the NRL for STEC (NRL-STEC), while for human samples this was done at the NRC for STEC (NRC-STEC). Detection of STEC (defined by the presence of *stx*) and determination of the O26, O103, O111, O145, and O157 serogroups in food and carcass swab samples was performed according to the International Organization for Standardization (ISO) Technical Specification (TS) 13136:2012 [21]. Isolation of *E. coli* O157 from food and carcass samples was performed according to ISO 16654:2001 [22]. A complete description for both ISO workflows applied during the initial investigation of both outbreaks can be found in the Supplementary Methods.

Detection and isolation of STEC in human stool samples at the time of the outbreak included selective growth on Sorbitol-MacConkey agar biplates with and without cefixime and tellurite ((CT)-SMAC; Lab M Limited, Lancashire, UK), followed by multiplex PCR detection of *stx1* (a, c, and d) and *stx2* (a–g), directly on the bacterial growth, as described previously [23,24,25]. Isolated human strains were confirmed as *E. coli* through biochemical identification tests (citrate, mobility indole urease, indole, urease, β-glucuronidase, and sorbitol), as previously described [26]. All food and carcass STEC O157 isolates were transferred from the NRL-STEC to NRC-STEC for further characterization and molecular comparison with human STEC O157. Shortly, serotyping was performed through slide-agglutination with O26, O103, O111, O121, and O145 antisera (Statens Serum Institut (SSI), Copenhagen, Denmark), supplemented with in-house O157 and H7 antisera for the isolates collected before 2013 (Limburg 2012 outbreak isolates), and with SSI O157 antisera and PCR detection of *fliC_H7_* [27] for the isolates collected in 2013 [28]. All (human, food, and carcass) isolates were further characterized through PCR, directly on the bacterial growth, for the detection of virulence genes (*stx*, *eae*, and *ehxA*), *stx* subtypes, and enteroaggregative *E. coli* virulence genes (*aaiC* and *aggR*), as previously described [23,24,29,30]. Human, food, and carcass swab isolates from both outbreaks were subjected to AMR susceptibility tests using the Kirby–Bauer disc diffusion method, according to European Committee on Antimicrobial Susceptibility Testing (EUCAST) recommendations (or Clinical Laboratory Standards Institute (CLSI) when no breakpoints were available from the former) for antibiotics selected by the Program for Food and Waterborne Disease and Zoonoses. In total, resistance against eleven antibiotics (I2A Diagnostics, Montpellier, France) was evaluated, distributed over six antibiotic drug groups, i.e., β-lactams (ampicillin and cefotaxime), aminoglycosides (streptomycin, kanamycin, and gentamycin), phenicols (chloramphenicol), trimethoprims (trimethoprim-sulfamethoxazole), sulfonamides (trimethoprim-sulfamethoxazole and sulfonamide), and tetracyclines (tetracycline). Insertion sequence 629-typing (IS629-typing), a fingerprinting technique based on the presence of IS629 throughout the STEC O157 genome, was routinely performed at the NRC-STEC to detect STEC O157 outbreak clusters and potentially identify already earlier circulating strains with identical and/or similar IS629 fingerprints. As part of the outbreak investigations, the food and carcass swab isolates were also typed with the IS629-typing method. Non-outbreak cases with similar or identical IS629-fingerprints to the outbreak isolates (indicated in red in Supplementary Materials, Table S1) were included in the phylogenetic study using pulsed-field gel electrophoresis (PFGE). IS629-typing and PFGE were conducted as described by Ooka et al. [5] and the PulseNet Standard Operating Procedure (SOP) for *E. coli* O157 [31,32], respectively, and analyzed using the BioNumerics software 6.5 (Applied Maths NV, bioMérieux, Sint-Martens-Latem, Belgium). Dendrograms were prepared with the unweighted pair group method with an arithmetic mean (UPGMA) for both PFGE (as part of the SOP) and IS629 profiles (because of its good performance).

### 2.2. Retrospective WGS-Based Outbreak Analysis

Both outbreaks were retrospectively analyzed with WGS. The applied WGS method is schematically depicted in Figure 1. The STEC O157 isolates with similar characteristics collected in the periods of both outbreaks were preserved in a glycerol–Brain–Heart infusion (BHI; Sigma-Aldrich, MO, USA) broth (40%) at −80 °C until the retrospective WGS-based analysis. All isolates were manipulated as previously described [33]. Shortly, total DNA (i.e., chromosomal and plasmid) was extracted using the GenElute Bacterial Genomic DNA kit (Sigma-Aldrich, MO, USA), with DNA elution in 10 mM Tris-HCl (pH 8.5). A random selection of 41 extra background isolates (Supplemental Table S1) was made, based on conventionally performed O157 serogrouping and detection of *stx* (i.e., STEC). The DNA of these isolates was prepared previously in the scope of another project using the DNeasy Blood and Tissue kit (Qiagen, Hilden, Germany) according to the manufacturer’s protocol, or the GenElute Bacterial Genomic DNA kit with DNA elution in 10 mM Tris-HCl (pH 8.5). Each DNA extract was preserved at −20 °C until Nextera XT DNA library preparation (Illumina, San Diego, CA, USA) according to the manufacturer’s instructions, and subsequently sequenced using a MiSeq instrument with the MiSeq V3 chemistry (Illumina, San Diego, CA, USA), for the production of 2 × 250 bp paired-end reads.

All applied analyses were performed using the tools with specific settings, as previously described [33]. Shortly, raw reads were trimmed with Trimmomatic 0.36 [34] and subsequently de novo assembled using SPAdes 3.13.0 [35]. Read-mapping based detection of genes associated with AMR genes, virulence genes, plasmid replicons, and serotype determining genes was performed using SRST2 0.2.0 [36] on the trimmed reads with the ResFinder [37,38], VirulenceFinder [9,38], PlasmidFinder [39], and SerotypeFinder [40] databases. Hits with <60% query coverage and >10% divergence (i.e., default settings) were omitted. Database sequences were retrieved from their corresponding sources on February 24th 2020, and clustered beforehand on 80% sequence identity using cd-hit 4.6.8 [41]. In silico core genome MLST (cgMLST) was performed through alignment of the assembled contigs using BLAST+ 2.6.0 [42,43] against the EnteroBase [44] cgMLST scheme containing 2513 loci (updated on February 24th, 2020). The relatedness between the isolates was then evaluated by constructing a minimum spanning tree based on the allele call matrix, using GrapeTree 1.5.0 [45] with the ‘method’ option set to ‘MSTreeV2’, and afterwards visualized using FigTree 1.4.3 [46]. The WGS data were deposited in the NCBI SRA repository [47] under BioProject numbers PRJNA574887 [33], PRJNA633966 [48], and PRJNA645975 (all in-house sequenced data). The accession numbers are listed in Supplemental Table S4 online.

## 3. Results

### 3.1. Description of the Selected Outbreaks and Isolates

To evaluate the potential benefits of WGS implementation in foodborne outbreak investigation for a smaller country, the two largest STEC outbreaks in Belgium over the past 10 years were selected for retrospective WGS-based analysis.

A first outbreak was situated in North-East Limburg, Belgium, in 2012, for which the applied conventional methods combined with the epidemiological data succeeded to detect the outbreak, define its cluster, and identify the causal source, resulting in outbreak management. The course of the outbreak was described in Braeye et al. [6]. In total, 21 and two STEC O157 isolates (Table 1) that were collected during and outside this outbreak, respectively, were included in the initial investigation. This outbreak was selected to evaluate whether WGS could have an added value in the investigation of such ‘textbook example’ foodborne outbreaks.

A second outbreak was situated throughout Flanders, Belgium, in 2013. Due to the lack of an epidemiological link between the human cases and the suspect food, in combination with minor discrepancies between applied conventional methods for the molecular comparison between the STEC O157 isolates (i.e., PFGE patterns and IS629 fingerprints), the outbreak could only partially be resolved. Indeed, no causal source with strong epidemiological evidence could be identified. The outbreak course was described earlier [49]. In total, 16 and four STEC O157 isolates with similar characteristics, i.e., the presence of *stx2a*, *eae*, and *ehxA* (Table 2) that were collected during and outside the outbreak period, respectively, were included in the initial outbreak investigation. The outbreak was selected to evaluate whether the increased resolution of WGS would offer an added value for its investigation.

Moreover, a selection of 41 earlier circulating STEC O157 background isolates (Supplemental Table S1) from different origin were included in the retrospective phylogenetic study of both outbreaks, in an attempt to put them within the context of STEC strains circulating in Belgium.

### 3.2. Comparing Isolate Characterization Using WGS and Conventional Methods

Both outbreaks were retrospectively analyzed with WGS, and characterization results were compared with those previously obtained with conventional methods (Limburg outbreak 2012: Table 1; Flanders outbreak 2013: Table 2).

Through WGS-based detection of the different O-and H-type determining gene alleles (Supplemental Table S2), all isolates of both outbreaks were identified as O157:H7, in agreement with the conventional serotyping results.

Moreover, using WGS data analysis, beside the *stx1* and/or *stx2* genes, in total 19 different virulence genes were detected that were present or partly present in each of the 37 outbreak isolates. In particular, per isolate, 17 virulence genes were additionally detected with WGS compared to those detected with conventional methods. Not only the genes, but also their specific alleles were identified with WGS (Supplemental Table S3).

The observed phenotypic AMR susceptibility patterns were correctly predicted from the AMR genotype obtained with WGS. Indeed, among the 37 outbreak isolates, the susceptibility tests indicated phenotypic AMR against antibiotic drug classes: (i) for the carcass isolate (TIAC1153, Table 1) against β-lactams (*bla_TEM-1B_*), aminoglycosides (*aph3′-Ia* and *strAB*), sulfonamides (*sul2*), tetracyclines (*tetA*), and trimethoprims (*dfrA8*); and (ii) for the human isolate (EH2285; Table 2) against β-lactams (*bla_TEM-1B_*), aminoglycosides (*aadA1* and *strAB*), tetracyclines (*tetA*), trimethoprims (*dfrA1*), and sulfonamides (*sul1* and *sul2*) for which WGS enabled the detection of the corresponding AMR genes (indicated between brackets). However, WGS-based AMR genotyping additionally detected the *mdfA* gene in all outbreak isolates, associated with resistance against macrolides according to the information provided by the ResFinder database [37,38]. However, MdfA has been reported as an *E. coli* multidrug transporter with an extraordinarily broad spectrum of drug recognition [50]. Since resistance against macrolides was not phenotypically tested in routine, correspondence with the full AMR phenotype could not be evaluated for this antibiotic group.

### 3.3. Comparison of the Phylogenetic Performance between WGS and Conventional Methods in Resolving Relatedness

The phylogenetic performances of conventional PFGE and IS629-typing applied during the former investigations, and WGS-based cgMLST-typing were compared for both outbreaks.

**Figure 2:**
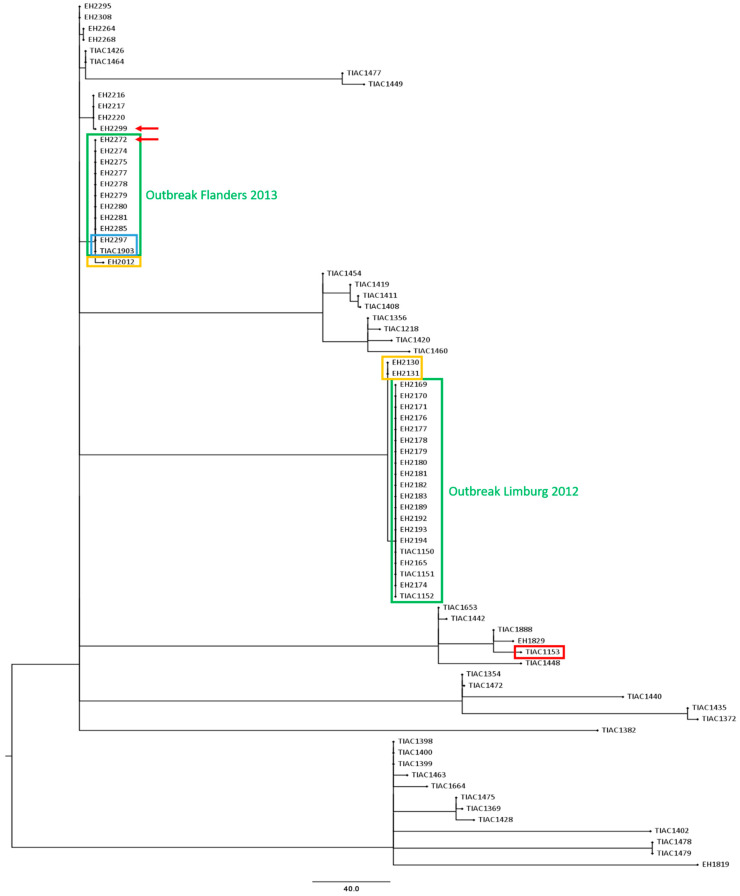
A cgMLST tree of all suspected outbreak isolates (Limburg 2012 and Flanders 2013) and the 41 background isolates. A minimum spanning tree was made based on the cgMLST allele matrices of all isolates using the MSTreeV2 method with GrapeTree. The outbreak clusters of Limburg 2012 and Flanders 2013 are outlined in green boxes. The cow carcass isolate that was discriminated from the Limburg 2012 outbreak cluster is visualized with a red box. Isolates that were identified with WGS to be closely related to the outbreak cluster are outlined in a yellow box. The two isolates of the 2013 outbreak in Flanders (EH2297 and TIAC1903) for which laboratory confirmation of the link was supported by epidemiological information are outlined in a blue box. Moreover, the isolates for which conventional PFGE and IS629 profiling was non-corresponding, are indicated with a red arrow. The scale bar represents the absolute number of cgMLST allele differences between isolates.

For the Limburg outbreak in 2012, all 17 human outbreak isolates had the exact same IS629 fingerprint and similar PFGE patterns (variation of one to two bands) compared to the three food isolates. Only the cow carcass isolate (TIAC1153) to which the meat was traced back had a different IS629 fingerprint [7] (Supplemental Figure S1), PFGE pattern [6,7], and virulence and AMR characteristics (both when determined with WGS and conventional methods) compared to all other outbreak isolates, and was therefore discriminated from the outbreak. Moreover, two extra human STEC O157 isolates (EH2130 and EH2131) collected outside the outbreak period were identified with identical IS629-fingerprints as the outbreak isolates (IS629-type F). An identical outbreak cluster was obtained with WGS-based cgMLST-typing (Figure 2). All human isolates were linked to the three food isolates, clustering together in a single clade carried by one single branch, and separated from the cow carcass isolate with 225 cgMLST alleles difference. Through cgMLST-typing of circulating background strains, the close positioning of the two extra human cases relative to the outbreak cluster (four cgMLST alleles difference, Figure 2: in a blue box) was determined.

**Figure 3:**
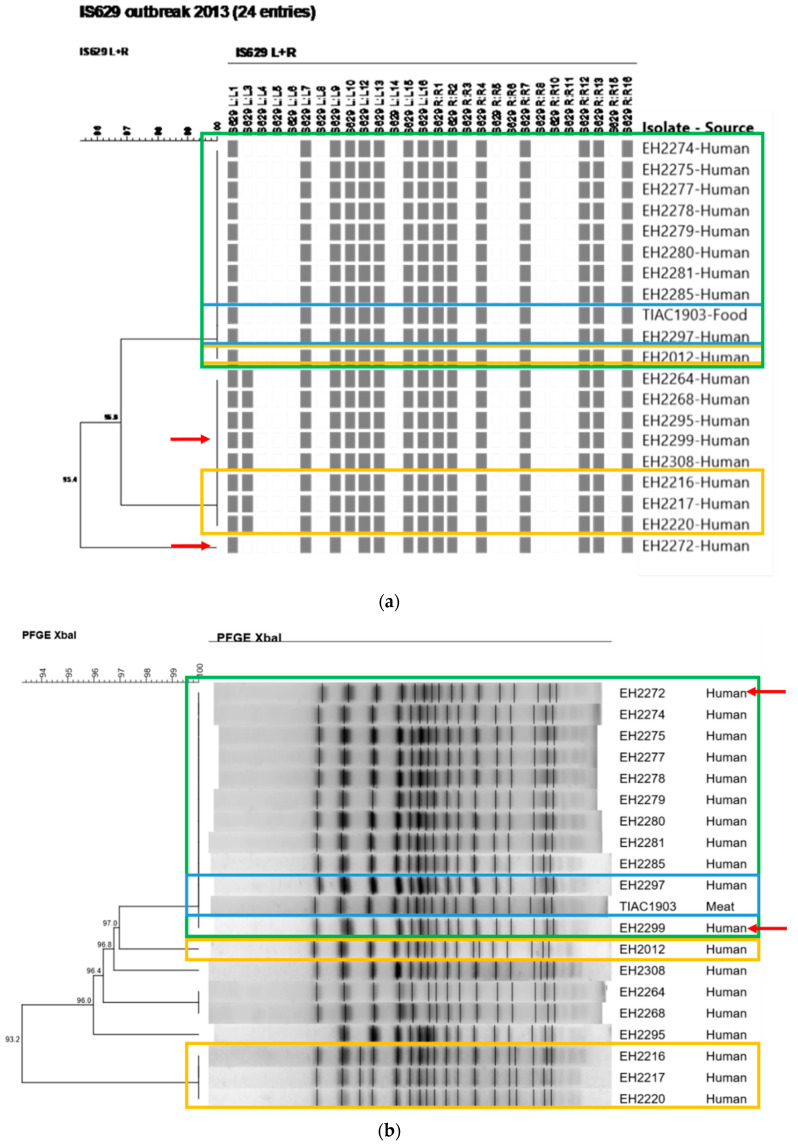
The IS629 fingerprints (**a**) and PFGE patterns (**b**) of all suspected isolates involved in the outbreak in Flanders (2013) are depicted. The scale bars represent the percentages of similarity between the isolates. All outbreak isolates clustering together (i.e., identical patterns) are outlined in a green box (identified as the outbreak cluster). The two isolates outlined in a blue box concern the human case for which the epidemiological link with the consumption of the contaminated food source (raw beef meat) was laboratory confirmed. Background isolates included in the initial outbreak investigation are outlined in a yellow box. Red arrows indicate the differences in the outbreak clusters obtained with both conventional methods.

During the initial investigation of the Flanders outbreak in 2013, undistinguishable or closely related PFGE and IS629 molecular typing profiles were observed between the STEC O157 isolates with similar virulence characteristics collected during the outbreak period (Figure 3). As part of the initial outbreak investigation, it was identified that four human STEC O157 background isolates had similar (EH2216, EH2217, and EH2220) and identical (EH2012) IS629 fingerprints compared to the investigated isolates collected during the outbreak period. These background isolates moreover had similar PFGE patterns (one or two bands different) compared to the isolates collected during the outbreak period (Figure 3: outlined in yellow boxes). When mutually comparing the PFGE-determined and IS629-determined outbreak clusters (Figure 3: outlined in green boxes), nine human isolates (EH2274, EH2275, EH2277, EH2278, EH2279, EH2280, EH2281, EH2285, and EH2297) had identical patterns compared to those of the food isolate (TIAC1903) with both methods. Only for isolate EH2297, the laboratory confirmed link was supported by epidemiological evidence for the suspected raw beef meat consumption (Figure 3: outlined in blue boxes). Four isolates (EH2264, EH2268, EH2295, and EH2308) were observed to have slightly different PFGE and IS629 patterns compared to their respective outbreak clusters. However, for two isolates (EH2272 and EH2299, Figure 3: red arrows) non-corresponding results were obtained between IS629-typing and PFGE, i.e., both isolates were considered as part of the PFGE-determined outbreak cluster, whereas IS629 fingerprints were divergent from the outbreak cluster. Consequently, the lack of epidemiological information concerning the patient’s diet history, in combination with the minor discrepancies observed between the conventional PFGE- and IS629-determined outbreak clusters, impeded cluster completion and, resolution of the outbreak with conventional methods. In contrast, cgMLST typing (Figure 2) enabled to clearly define the outbreak cluster. Ten human isolates (EH2272, EH2274, EH2275, EH2277, EH2278, EH2279, EH2280, EH2281, EH2285, and EH2297) clustered together with the food isolate (TIAC1903) in a single clade with zero cgMLST allele differences. Moreover, the relatedness of the two human isolates collected during the outbreak period (EH2272 and EH2299), for which PFGE and IS629 positioned them divergently relative to the outbreak cluster, was resolved with cgMLST typing. Indeed, one isolate (EH2272) was placed together on the same branch with all other outbreak isolates (zero cgMLST allele differences), while the other (EH2299) was discriminated from the outbreak cluster with 12 cgMLST allele differences. As also suggested by PFGE- and IS629-typing, cgMLST typing clearly separated four human isolates that were collected during the outbreak period (EH2264, EH2268, EH2295, and EH2308) from the outbreak cluster, i.e., 6–8 identified cgMLST allele differences between the cases and the outbreak cluster. Moreover, a background isolate (EH2012) was positioned closely to the cgMLST-determined outbreak cluster, i.e., two cgMLST loci with different alleles, whereas identical molecular patterns compared to the outbreak cluster were only identified by conventional IS629-typing. Additionally, human isolate EH2285 that was determined to be part of the outbreak cluster, showed genotypic and phenotypic resistance to several antibiotics (ampicillin, streptomycin, tetracycline, and trimethoprim-sulfamethoxazole, Table 2). However, no AMR was detected for all other cgMLST-determined phylogenetically linked isolates in the same outbreak cluster. Interestingly, compared to the other isolates within the outbreak cluster, two extra plasmid replicons (IncI1-Iγ and IncQ1, Table 2) were detected. Moreover, when aligning the assembled contigs against the PlasmidFinder and ResFinder databases, it was observed that the majority (6/8) of the AMR genes (*aadA1, strA, strB, dfrA1, sul1,* and *sul2*) was detected across the full length of the contig also containing the IncQ1 plasmid replicon. These results suggest that isolate EH2285 gained an AMR-genes containing plasmid.

## 4. Discussion

Our study demonstrated that WGS-based cgMLST typing delivered a higher discriminatory power compared to conventional methods, enabling more accurate phylogenetic analysis, as similarly reported before [4,51,52,53,54,55]. This increased resolution was clearly expressed in the retrospective investigation of the Flanders 2013 outbreak. If WGS was available and applied at that time, it would have enabled quick and unambiguous outbreak cluster definition. Moreover, the presence of background isolates in the cgMLST tree allowed putting the relationship between isolates collected during the outbreak in the perspective of circulating background strains, which enabled outbreak cluster delineation. Nevertheless, as no epidemiologically linked food isolate was obtained, WGS would not have resolved the 2013 outbreak either. Of particular interest was, however, that the presence of these background isolates in the cgMLST-based phylogeny enabled the direct and accurate identification of human background isolates as closely related to the outbreak clusters. Importantly, this identification remained unclear with conventional methods, and was only suggested with IS629 fingerprinting. Interestingly, these cases were reported 3.5 months and 2.5 years before the start of the Limburg 2012 and Flanders 2013 outbreaks, respectively, indicating that this strain was already circulating before. Having this reliable knowledge during the initial investigation could have further directed epidemiological investigations to find the source of infection, especially for the Flanders 2013 outbreak. Although WGS is gradually being introduced in European national public health agencies [12], i.e., for human isolates, this clearly highlights the need for its full implementation in food safety surveillance systems as well, to place outbreaks within the context of historical and/or circulating background strains of different origins. This would potentially enable instant detection of a suspect source through its clustering with human cases, which can stimulate in-depth epidemiological investigations, especially when considering already circulating strains obtained via routine foodborne pathogen surveillance. Consequently, outbreaks can potentially be managed or prevented before they even started [11]. As successfully applied by, e.g., the GenomeTrakr Network [56] and as also demonstrated before [8], through this approach, routine implementation of WGS in both food safety and public health practices enables one to improve foodborne outbreak investigations. It is, therefore, crucial that national policy makers of smaller and/or less developed countries are convinced to financially support WGS implementation in both public health and food safety resources, including surveillance.

Another important benefit of WGS-based outbreak investigation is the all-in-one workflow, with less hands-on-time (requiring five laboratory working days, i.e., three days for isolation and two days for sample preparation). The sequencing process itself takes approximately two days without hands-on-time. In contrast, conventional methods consist of labor-intensive sequential culturing and analyses steps (approximately four working days, and three additional with PFGE). Moreover, extra cost and time should be considered for: (i) isolate transport to the NRC-STEC for phylogenetic evaluation, in contrast to rapid and cost-free WGS data sharing (once the data infrastructure installed); and (ii) extra sequential analyses to retrieve an equally complete characterization as with WGS. Taking this into account would render WGS also cost- and time-efficient. To further improve its timeliness, real-time data analysis [57] reducing the sequencing time, or metagenomics [58,59] eliminating the isolation time, can be applied.

In comparison to conventional methods, WGS enables the detection of most serotypes (some serogroups share identical O-antigen determining genes [60]), and the complete virulence genotype. Indeed, routinely determined characteristics for food STEC isolates in various European NRLs were, and still are, limited to those evaluated in the ISO/TS 13136:2012 standard [21], i.e., *stx1*, *stx2*, and *eae* virulence genes, and European “top five” serogroups (O26, O103, O111, O145, and O157) [61]. Solely for the purpose of the outbreak investigations, food STEC isolates were characterized similarly as human STEC through the additional detection of the virulence *ehxA*, *aaiC*, and *aggR* genes, *stx* subtypes (*stx1*(a,c,d) and *stx2*(a-g)), the O121 serogroup, and the H7-type. It should be noted that this in-depth STEC characterization is currently done by only a very limited number of EU clinical laboratories. Detection of these genes and serogroups was motivated by the initial assumption that only STEC with these characteristics correlate to severe disease [24,62,63,64,65,66]. However, recent literature has revised this hypothesis by determining that all STEC, regardless of serotype, *stx* subtype, and the presence/absence of these virulence genes, are capable of causing severe disease [61,67,68,69,70,71]. This suggests an important role of additional virulence genes or specific variants in pathogenicity. It moreover indicates that a more holistic approach for STEC characterization, including *stx* and other virulence gene subtyping, is required, in particular to comprehend and predict the pathogenic potential of STEC in humans. From this point of view, ISO/TS 13136 is being revised to add steps for more complete STEC characterization, with the advice to apply WGS [61].

Through AMR genotyping, WGS has the potential to predict the corresponding AMR phenotype. Although antibiotic treatment is controversial for STEC infections [72,73], WGS-based AMR prediction can be valuable for treatment choice in other foodborne diseases, and contains important information for surveillance. Notwithstanding the limited phenotypic AMR observed within this study, WGS-based genotypic AMR prediction was fully compliant with the phenotypic AMR. Indeed, multiple studies have demonstrated high concordance between AMR susceptibility testing and WGS-based phenotype prediction for STEC (97%) [74,75,76]. In contrast to conventional testing that is targeted to specific antibiotics, WGS enables full AMR phenotype prediction. In fact, resistance against macrolides in all outbreak isolates was only predicted with WGS, while left unknown with routine testing, because it was not evaluated. Interestingly, WGS offers the possibility to suggest and potentially determine the location of AMR genes, i.e., chromosomal or on a plasmid. Indeed, despite long read sequencing still being a prerequisite for these purposes, the gain of an AMR-containing plasmid was suggested for the 2013 Flanders outbreak isolate EH2285. This knowledge is valuable, especially for risk assessment of the spread of AMR. Additionally, this also highlighted that despite having an identical cgMLST-type compared to other outbreak isolates, still some characteristics can differ. We chose cgMLST to determine relationships, since this method is currently applied by the Belgian NRC-STEC for the phylogenetic analyses of human isolates. Moreover, cgMLST has gained interest with regard to the transferability of its standardized scheme, which allows outbreaks to be analyzed and compared across laboratories [54,55]. WGS can, however, provide ultimate resolution through single nucleotide polymorphism (SNP) typing approaches, which might further fine-tune the clusters observed with cgMLST-typing in this study. If the differentiating characteristics are, however, all localized on a plasmid, these methods should take this into account to allow an increased discrimination.

Despite the demonstrated benefits, some bottlenecks remain that should be addressed to enable WGS’s full exploitation. There is still a need for a harmonized use of defined standards concerning DNA quality, WGS data quality, and analysis, to allow reliable data exchange between (inter)national laboratories [77]. Consequently, several studies and the ISO TC34-SC9-WG25 have evaluated wet-lab and dry-lab workflows [76,78,79,80,81], and are preparing general guidance, respectively, for WGS and subsequent foodborne pathogen characterization and typing. Moreover, quality assurance systems should be put in place that guarantee data accuracy, as these will be used to make crucial, sometimes even legal, decisions in food safety management. The outsourcing and centralization of all WGS activities to large qualified centers in Europe could contribute to the harmonization of WGS workflows, and simultaneously allow smaller and/or developing countries with less resources to perform WGS. However, by doing so, crucial time during outbreak investigations can be lost because of isolate transfer. Moreover, issues related to sensitive metadata protection, with possible legal/political consequences, rights for data sharing with the original laboratory requesting the analysis, and use of the generated data without proper recognition, should also be considered. Indeed, successful WGS application is additionally dependent on data exchange between food safety and public health laboratories, and, with regard to One Health initiatives, all different sectors, including animal and environmental [82]. Currently, these data are shared ad hoc between the Belgian NRLs and NRC, when epidemiological evidence for an outbreak exists and budget for WGS-based outbreak investigation is available. To facilitate data storage and support a rapid response to food safety incidents, a repository in which WGS data and corresponding metadata of isolates from different sectors and countries are centralized, is highly required. Hurdles that come with their accessibility and the protection of sensitive metadata are being addressed internationally, however, still require some discussion at the European level. Publicly open repositories [83], such as the GenomeTrakr database [56,84], create the opportunity for international laboratories to participate in data sharing initiatives. Moreover, it includes data from different sectors (food, environment, and clinical) and provides updated phylogenetic trees on a daily basis. In contrast, EFSA and ECDC are in favor of the development of a closed access repository to protect the confidentiality of data [16,18]. However, limited accessibility of databases also impacts the diversity in submitted isolates. Subsequently, this will directly interfere with the potential to detect genomics-based matches of human clusters with food and/or environmental isolates, but also to identify genomics-based matches with historical cases and/or already earlier circulating strains. Ideally, WGS data should be deposited in an openly accessible way, at a real-time basis, such as proposed by the Global Microbial Identifier (GMI) initiative [85]. However, the question is whether this open data availability will not frighten the food industry operators to participate in routine WGS application for surveillance, since it might affect the credibility of the respective institution/plant dealing with (supposed) contamination. This will even be more of an issue when contaminations are traced back to outbreaks without solid epidemiological evidence, for which the power of WGS risks to tempt to. Nevertheless, complete food safety management is based on the participation of all players in the field, including the food industry. This is also important in the sampling strategy. Indeed, the extensiveness and effectiveness of centralized repositories depend on the maturity of national surveillance systems. Especially in smaller countries, such as Belgium, surveillance policies indirectly suffer from the sometimes complex political structures and limited funding [14,15]. European countries are obliged to comply with European regulation (2073/2005 and amendments) and to investigate the presence of STEC in foods and animals according to directive 2003/99/EC. For the latter, country-specific sampling legislations often exist, but since the directive is not explicit, this results in a rather biased sampling. Additionally, depending on the pathogen, isolation from food matrices can be difficult using ISO methods. This impedes further characterization, and leads to a shift towards molecular detection solely. As a result, only few food isolates are eventually received/obtained to be analyzed, especially in smaller countries. Subsequently, a vicious circle arises from the perspective that investment in WGS is not of interest for such limited amount of samples. Increasing this is possible through revising sampling strategies (and regulation) by targeting an increased variety of food and/or environmental samples [86], and through inciting food operators to send samples to the NRL for analysis. To facilitate the identification of small local, to large multi-country outbreaks that often remain undetected with general surveillance systems [1], the development of sampling strategies at the local, national, and international level might help. However, these revisions will again rely on financial resources. Although the cost of capacity building in NRLs of smaller countries is perceived as a major limiting factor, its added value for outbreak investigation has been clearly demonstrated for STEC, as well as for other foodborne pathogens [53,54,87,88], rendering WGS a cost-effective approach. For example, a Canadian analysis of the impact of WGS implementation in *Salmonella* surveillance reported a yearly net economic benefit of $ 90.25 million CAD or € 59.57 million for salmonellosis solely [89]. Although country-specific, these data suggest a logarithmic equivalent economic benefit for *Salmonella* as well as for other foodborne pathogens [83], even in smaller countries. As the use of WGS in food surveillance would allow rapidly picking up signals, thereby preventing outbreaks, it will have clearly both health and economic benefits. WGS could increase confidence in the food produced in the country that installed such a surveillance system, which is important for international trade. Moreover, the eventual costs linked to contaminations in the food sector, could be avoided, and will most likely exceed those linked to WGS investments. Therefore, as routine WGS implementation in surveillance would be of financial interest to the food business operators themselves, it could be suggested that part of the required costs are covered by them.

Conclusively, although some national and international bottlenecks remain, this study demonstrated that even for NRLs in smaller countries, WGS has clear benefits over conventional methods for foodborne outbreak investigation. However, its benefits will only be fully exploited if a sufficient budget is made available to ensure its implementation in surveillance systems as well. Indeed, feeding the database of circulating and/or historical background strains could direct further epidemiological inquiries in foodborne outbreak investigation, and could allow early warning. With this study, we intend to encourage policy makers to financially support WGS implementation as the new standard for food safety, highly needed within an international context.

## Figures and Tables

**Table 1:** The characteristics of, and relatedness between the Limburg 2012 outbreak isolates, determined with conventional methods and WGS data analysis.

Isolate Reference	Isolation Date (dd/mm/yyyy)	Origin	Conventional Methods									WGS																									
			Serotype	Virulence Genes						AMR	Relation to Outbreak	Serotype	Virulence Genotype																	AMR Genotype							Relation to Outbreak
				*stx1*	*stx2*	*eae*	*ehxA*	*aggR*	*aaiC*				*stx1*	*stx2*	*eae*	*ehxA*	aggR	*aaiC*	*katP*	*toxB*	*astA*	*esp*	*gad*	*nle*	*tir*	*iss*	*etpD*	*iha*	*tccP*	*bla_TEM-1B_*	*aph(3’)-Ia*	*str*	*sul2*	*tetA*	*dfrA8*	*mdfA*	
**EH2165**	06/06/2012	Human	O157:H7	a	a	+	+	-	-	S	O	O157:H7	a	a	+	+	-	-	+	+	+	A, B, F, J, P	+	A, B, C	+	+	+	+	+	-	-	-	-	-	-	+	O
**EH2169**	07/06/2012	Human	O157:H7	a	a	+	+	-	-	S	O	O157:H7	a	a	+	+	-	-	+	+	+	A, B, F, J, P	+	A, B, C	+	+	+	+	+	-	-	-	-	-	-	+	O
**EH2170**	12/06/2012	Human	O157:H7	a	a	+	+	-	-	S	O	O157:H7	a	a	+	+	-	-	+	+	+	A, B, F, J, P	+	A, B, C	+	+	+	+	+	-	-	-	-	-	-	+	O
**EH2171**	13/06/2012	Human	O157:H7	a	a	+	+	-	-	S	O	O157:H7	a	a	+	+	-	-	+	+	+	A, B, F, J, P	+	A, B, C	+	+	+	+	+	-	-	-	-	-	-	+	O
**EH2174**	12/06/2012	Human	O157:H7	a	a	+	+	-	-	S	O	O157:H7	a	a	+	+	-	-	+	+	+	A, B, F, J, P	+	A, B, C	+	+	+	+	+	-	-	-	-	-	-	+	O
**EH2176**	08/06/2012	Human	O157:H7	a	a	+	+	-	-	S	O	O157:H7	a	a	+	+	-	-	+	+	+	A, B, F, J, P	+	A, B, C	+	+	+	+	+	-	-	-	-	-	-	+	O
**EH2177**	08/06/2012	Human	O157:H7	a	a	+	+	-	-	S	O	O157:H7	a	a	+	+	-	-	+	+	+	A, B, F, J, P	+	A, B, C	+	+	+	+	+	-	-	-	-	-	-	+	O
**EH2178**	13/06/2012	Human	O157:H7	a	a	+	+	-	-	S	O	O157:H7	a	a	+	+	-	-	+	+	+	A, B, F, J, P	+	A, B, C	+	+	+	+	+	-	-	-	-	-	-	+	O
**EH2179**	14/06/2012	Human	O157:H7	a	a	+	+	-	-	S	O	O157:H7	a	a	+	+	-	-	+	+	+	A, B, F, J, P	+	A, B, C	+	+	+	+	+	-	-	-	-	-	-	+	O
**EH2180**	14/06/2012	Human	O157:H7	a	a	+	+	-	-	S	O	O157:H7	a	a	+	+	-	-	+	+	+	A, B, F, J, P	+	A, B, C	+	+	+	+	+	-	-	-	-	-	-	+	O
**EH2181**	14/06/2012	Human	O157:H7	a	a	+	+	-	-	S	O	O157:H7	a	a	+	+	-	-	+	+	+	A, B, F, J, P	+	A, B, C	+	+	+	+	+	-	-	-	-	-	-	+	O
**EH2182**	14/06/2012	Human	O157:H7	a	a	+	+	-	-	S	O	O157:H7	a	a	+	+	-	-	+	+	+	A, B, F, J, P	+	A, B, C	+	+	+	+	+	-	-	-	-	-	-	+	O
**EH2183**	19/06/2012	Human	O157:H7	a	a	+	+	-	-	S	O	O157:H7	a	a	+	+	-	-	+	+	+	A, B, F, J, P	+	A, B, C	+	+	+	+	+	-	-	-	-	-	-	+	O
**EH2189**	22/06/2012	Human	O157:H7	a	a	+	+	-	-	S	O	O157:H7	a	a	+	+	-	-	+	+	+	A, B, F, J, P	+	A, B, C	+	+	+	+	+	-	-	-	-	-	-	+	O
**EH2192**	26/06/2012	Human	O157:H7	a	a	+	+	-	-	S	O	O157:H7	a	a	+	+	-	-	+	+	+	A, B, F, J, P	+	A, B, C	+	+	+	+	+	-	-	-	-	-	-	+	O
**EH2193**	26/06/2012	Human	O157:H7	a	a	+	+	-	-	S	O	O157:H7	a	a	+	+	-	-	+	+	+	A, B, F, J, P	+	A, B, C	+	+	+	+	+	-	-	-	-	-	-	+	O
**EH2194**	02/07/2012	Human	O157:H7	a	a	+	+	-	-	S	O	O157:H7	a	a	+	+	-	-	+	+	+	A, B, F, J, P	+	A, B, C	+	+	+	+	+	-	-	-	-	-	-	+	O
**TIAC1150**	27/06/2012	Filet americain	O157:H7	a *	a *	+	+ *	-	-	S *	O	O157:H7	a	a	+	+	-	-	+	+	+	A, B, F, J, P	+	A, B, C	+	+	+	+	+	-	-	-	-	-	-	+	O
**TIAC1151**	19/06/2012	Filet americain	O157:H7	a *	a *	+	+ *	-	-	S *	O	O157:H7	a	a	+	+	-	-	+	+	+	A, B, F, J, P	+	A, B, C	+	+	+	+	+	-	-	-	-	-	-	+	O
**TIAC1152**	19/06/2012	Filet americain	O157:H7	a *	a *	+	+ *	-	-	S *	O	O157:H7	a	a	+	+	-	-	+	+	+	A, B, F, J, P	+	A, B, C	+	+	+	+	+	-	-	-	-	-	-	+	O
**TIAC1153**	18/06/2012	Carcass swab	O157:H7	a *	c *	+	+ *	-	-	AMP, KAN, STR, SUL, SXT, TET *	N	O157:H7	a	c	+	+	-	-	+	+	+	A, B, F, J, P	+	A, B, C	+	+	+	+	+	+	+	A, B	+	+	+	+	N

All STEC O157 isolates with similar characteristics that were collected during the Limburg outbreak period in 2012 are shown. Isolates originating from food matrices and swabs are indicated with a TIAC-number, and isolates originating from human stools are indicated with an EH-number. The genotypic characteristics of all isolates with the conventional methods (serotyping and virulence gene detection), and WGS data analysis (serotyping, virulence genotyping, AMR genotyping using SRST2 for the SerotypeFinder, VirulenceFinder, and ResFinder databases, respectively) are visually indicated in green (+; present), or red (−; absent). Moreover, with WGS, the presence of plasmid replicons IncFIB and IncFII was detected in all isolates (except for isolate TIAC1153 for which the IncQ1 replicon was detected additionally) using SRST2 with the PlasmidFinder database. The detected subtype of the *stx* gene is specified. For the *esp* and *nle* operons, the constituting detected genes are specified. The detected AMR genes correspond to resistance against the following antibiotic drug classes: β-lactams (*bla_TEM-1B_)*, aminoglycosides (*aph(3’)-Ia* and *strAB*), trimethoprims (*dfrA8*), sulfonamides (*sul2*), tetracyclines (*tetA*), and macrolides (*mdfA*). Phenotypic AMR susceptibility is indicated as S, whereas resistance is indicated with the abbreviation of the respective antibiotic (AMP: Ampicillin, KAN: Kanamycin, STR: Streptomycin, SUL: Sulfonamides, SXT: Trimethoprim-Sulfamethoxazole, and TET: Tetracycline). The *stx2* subtype of the carcass isolate TIAC1153 (*stx2c*), identified with the conventional and WGS analysis, differs from the *stx2* subtype of the other outbreak isolates (*stx2a*). It is indicated whether the isolates were considered as part of the outbreak (O; conventional IS629-type: F) or as unrelated to the outbreak (non-outbreak, N; conventional IS629-type: T) according to the analysis with both approaches. Knowledge on genes/subtypes, and the AMR phenotype in/of the food and carcass isolates (which was not determined according to ISO/TS 13136:2012, and ISO 16654:2001) indicated with a star (*), was only obtained because the isolates were transferred to the NRC-STEC for further characterization, according to the conventional workflow routinely applied on human isolates. Although H7-typing in food isolates is not obliged according to ISO/TS 13136:2012, it was routinely determined by the NRL-STEC through the GeneDisc applied as part of the ISO (see Supplementary Methods). Moreover, this H7-type was also determined at the NRC-STEC through slide agglutination.

**Table 2:** The characteristics of and relatedness between the isolates with similar characteristics collected during the Flanders 2013 outbreak period, determined with conventional methods and WGS data analysis.

Isolate Reference	Isolation Date (dd/mm/yyyy)	Origin	Conventional Methods										WGS																								
			Serotype	Virulence Genes						AMR	Relation to Outbreak	Serotype	Virulence Genotype																	AMR Genotype							Relation to Outbreak
				*stx1*	*stx2*	*eae*	*ehxA*	*aggR*	*aaiC*				*stx1*	*stx2*	*eae*	*ehxA*	aggR	*aaiC*	*katP*	*toxB*	*astA*	*esp*	*gad*	*nle*	*tir*	*iss*	*etpD*	*iha*	*tccP*	*bla_TEM-1B_*	*aadA1*	*str*	*tetA*	*dfrA1*	*sul*	*mdfA*	
**EH2264**	15/05/2013	Human	O157:H7	-	a	+	+	-	-	S	N	O157:H7	-	a	+	+	-	-	+	+	+	A, B, F, J, P	+	A, B, C	+	+	+	+	+	-	-	-	-	-	-	+	N
**EH2268**	28/05/2013	Human	O157:H7	-	a	+	+	-	-	S	N	O157:H7	-	a	+	+	-	-	+	+	+	A, B, F, J, P	+	A, B, C	+	+	+	+	+	-	-	-	-	-	-	+	N
**EH2272**	08/06/2013	Human	O157:H7	-	a	+	+	-	-	S	?	O157:H7	-	a	+	+	-	-	+	+	+	A, B, F, J, P	+	A, B, C	+	+	+	+	+	-	-	-	-	-	-	+	O *
**EH2274**	13/06/2013	Human	O157:H7	-	a	+	+	-	-	S	O*	O157:H7	-	a	+	+	-	-	+	+	+	A, B, F, J, P	+	A, B, C	+	+	+	+	+	-	-	-	-	-	-	+	O *
**EH2275**	13/06/2013	Human	O157:H7	-	a	+	+	-	-	S	O *	O157:H7	-	a	+	+	-	-	+	+	+	A, B, F, J, P	+	A, B, C	+	+	+	+	+	-	-	-	-	-	-	+	O *
**EH2277**	18/06/2013	Human	O157:H7	-	a	+	+	-	-	S	O *	O157:H7	-	a	+	+	-	-	+	+	+	A, B, F, J, P	+	A, B, C	+	+	+	+	+	-	-	-	-	-	-	+	O *
**EH2278**	18/06/2013	Human	O157:H7	-	a	+	+	-	-	S	O *	O157:H7	-	a	+	+	-	-	+	+	+	A, B, F, J, P	+	A, B, C	+	+	+	+	+	-	-	-	-	-	-	+	O *
**EH2279**	18/06/2013	Human	O157:H7	-	a	+	+	-	-	S	O *	O157:H7	-	a	+	+	-	-	+	+	+	A, B, F, J, P	+	A, B, C	+	+	+	+	+	-	-	-	-	-	-	+	O *
**EH2280**	18/06/2013	Human	O157:H7	-	a	+	+	-	-	S	O *	O157:H7	-	a	+	+	-	-	+	+	+	A, B, F, J, P	+	A, B, C	+	+	+	+	+	-	-	-	-	-	-	+	O *
**EH2281**	20/06/2013	Human	O157:H7	-	a	+	+	-	-	S	O *	O157:H7	-	a	+	+	-	-	+	+	+	A, B, F, J, P	+	A, B, C	+	+	+	+	+	-	-	-	-	-	-	+	O *
**EH2285**	28/06/2013	Human	O157:H7	-	a	+	+	-	-	AMP, STR, TET, SXT	O *	O157:H7	-	a	+	+	-	-	+	+	+	A, B, F, J, P	+	A, B, C	+	+	+	+	+	+	+	A, B	+	+	1, 2	+	O *
**EH2295**	12/07/2013	Human	O157:H7	-	a	+	+	-	-	S	N	O157:H7	-	a	+	+	-	-	+	+	+	A, B, F, J, P	+	A, B, C	+	+	+	+	+	-	-	-	-	-	-	+	N
**EH2297**	16/07/2013	Human	O157:H7	-	a	+	+	-	-	S	O	O157:H7	-	a	+	+	-	-	+	+	+	A, B, F, J, P	+	A, B, C	+	+	+	+	+	-	-	-	-	-	-	+	O
**EH2299**	18/07/2013	Human	O157:H7	-	a	+	+	-	-	S	?	O157:H7	-	a	+	+	-	-	+	+	+	A, B, F, J, P	+	A, B, C	+	+	+	+	+	-	-	-	-	-	-	+	N
**EH2308**	31/07/2013	Human	O157:H7	a	a	+	+	-	-	S	N	O157:H7	a	a	+	+	-	-	+	+	+	A, B, F, J, P	+	A, B, C	+	+	+	+	+	-	-	-	-	-	-	+	N
**TIAC1903**	01/07/2013	Filet americain	O157:H7	-	a *	+	+ *	- *	- *	S *	O	O157:H7	-	a	+	+	-	-	+	+	+	A, B, F, J, P	+	A, B, C	+	+	+	+	+	-	-	-	-	-	-	+	O

All STEC O157 isolates with similar characteristics collected during the Flanders outbreak period in 2013 are shown. Isolates originating from food matrices are indicated with a TIAC-number, and isolates originating from human stools are indicated with an EH-number. The genotypic characteristics of all isolates with the conventional methods (serotyping and virulence gene detection), and WGS data analysis (serotyping, virulence genotyping, AMR genotyping, plasmid replicon detection using SRST2 for the SerotypeFinder, VirulenceFinder, ResFinder, and PlasmidFinder databases, respectively) are visually indicated in green (+; present), or red (−; absent). The detected subtype of the *stx* gene is specified. For the *esp* and *nle* operons, the constituting detected genes are specified. For the human isolate (EH2308), *stx1a* could be detected additionally to *stx2a* using both the conventional and the WGS method. The detected AMR genes correspond to resistance against the following antibiotic drug classes: β-lactams (*bla_TEM-1B_*), aminoglycosides (*aadA1* and *strAB*), trimethoprims (*dfrA1*), sulfonamides (*sul1* and *sul2*), tetracyclines (*tetA*), and macrolides (*mdfA*). Phenotypic AMR susceptibility is indicated as S, whereas resistance is indicated with the abbreviation of the respective antibiotic (AMP: Ampicillin, STR: Streptomycin, TET: Tetracycline, and SXT: Trimethoprim-Sulfamethoxazole). With conventional methods, it was indicated whether the isolates were considered as: (i) molecularly indistinguishable (O *; IS629-type B1), however, without epidemiological support; (ii) molecularly indistinguishable (O; IS629-type B1) with epidemiological support; (iii) uncertain molecular relation to the suspect food (non-corresponding PFGE and IS629 patterns) not supported by epidemiological data (“?”; IS629-types B2 and B3); and (iv) probably molecularly unrelated to the suspect food (N; IS629-type B2) not supported by epidemiological evidence. With WGS-based cgMLST typing, it was indicated whether the isolates were considered as: (i) related to the outbreak (O *), however, without epidemiological support; (ii) related to the outbreak (O) with epidemiological support; and (iii) unrelated to the outbreak (non-outbreak, N). Knowledge on genes/subtypes, and the AMR phenotype in/of the food isolate (which was not determined according to ISO/TS 13136:2012, and ISO 16654:2001) indicated with a star (*), was only obtained because the isolate was transferred to the NRC-STEC for further characterization according to the conventional workflow routinely applied on human isolates. Although H7-typing in food isolates is not obliged according to ISO/TS 13136:2012, it was routinely determined by the NRL-STEC through the GeneDisc applied as part of the ISO (see Supplementary Methods). Moreover, this H7-type was also determined at the NRC-STEC through the PCR.

## Supplementary Materials

The following are available online at https://www.mdpi.com/2304-8158/9/8/1030/s1, Table S1: Characteristics of all background isolates determined with conventional methods and WGS, Table S2: WGS-based serotype determination, Table S3: WGS-based virulence allele typing, Table S4: NCBI SRA accession numbers of WGS data of all isolates, Figure S1: Conventional typing results of the Limburg 2012 outbreak, Methods: Conventional ISO workflows for STEC detection, isolation and identification from food samples, as performed during the initial outbreak investigation.
