# Supplementary material for: The Benefits of Whole Genome Sequencing for Foodborne Outbreak Investigation from the Perspective of a National Reference Laboratory in a Smaller Country
Source: Foods. 2020 Aug 1;9(8):1030. doi: 10.3390/foods9081030 (PMC7466227; doi:10.3390/foods9081030)
Supplement: Supplementary file 1 [file foods-09-01030-s001.pdf]

foods

MDPI

Article

The benefits of whole genome sequencing for foodborne outbreak investigation from a National Reference Laboratory perspective in a smaller country

Stéphanie Nouws[1,2], Bert Bogaerts[1,2], Bavo Verhaegen[3], Sarah Denayer[3], Florence Crombé[4], Klara De Rauw[4], Denis Piérard[4], Kathleen Marchal[2,5,6], Kevin Vanneste[1], Nancy H.C. Roosens[1], and Sigrid C.J. De Keersmaecker[1,*]

[1] Department of Expertise and service provision, Transversal activities in Applied Genomics, Sciensano, 1050 Brussels, Belgium; Stephanie.Nouws@Sciensano.be (S.N.), Bert.Bogaerts@Sciensano.be (B.B.), Kevin.Vanneste@Sciensano.be (K.V.), Nancy.Roosens@Sciensano.be (N.R.), and Sigrid.DeKeersmaercker@Sciensano.be (S.D.K.)

[2] Department of Information Technology, IDLab, imec, Ghent University, 9052 Ghent, Belgium; Kathleen.Marchal@ugent.be (K.M.)

[3] National Reference Laboratory for Shiga toxin-producing *Escherichia coli* (NRL-STEC), National Reference Laboratory for Foodborne Outbreaks (NRL-FBO); Department of Infectious diseases in humans, Foodborne Pathogens, Sciensano, 1050 Brussels, Belgium; Bavo.Verhaegen@Sciensano.be (B.V.), and Sarah.Denayer@Sciensano.be (S.D.)

[4] Vrije Universiteit Brussel (VUB), Universitair Ziekenhuis Brussel (UZ Brussel), Department of Microbiology and Infection Control, National Reference Center for Shiga toxin-producing *Escherichia coli* (NRC-STEC), 1090 Brussels, Belgium; Florence.Crombe@uzbrussel.be (F.C.), Klara.DeRauw@biomerieux.com (K.D.R.), and Denis.Pierard@uzbrussel.be (D.P.)

[5] Department of Plant Biotechnology and Bioinformatics, Ghent University, 9052 Ghent, Belgium; Kathleen.Marchal@ugent.be (K.M.)

[6] Department of Genetics, University of Pretoria, 0083 Pretoria, South-Africa; Kathleen.Marchal@ugent.be (K.M.)

* Correspondence: Sigrid.Dekeersmaecker@Sciensano.be; Tel.: +32-2-642-5257 (S.D.K.)

Received: date; Accepted: date; Published: date

Abstract: Gradually, conventional methods for foodborne pathogen typing are replaced by whole genome sequencing (WGS). Despite studies describing the overall benefits, National Reference Laboratories of smaller countries often show slower uptake of WGS, mainly because of significant investments required to generate and analyze data of a limited amount of samples. To facilitate this process and incite policy makers to support its implementation, a Shiga toxin-producing *Escherichia coli* (STEC) O157:H7 (*stx1*+, *stx2*+, *eae*+) outbreak (2012) and a STEC O157:H7 (*stx2*+, *eae*+) outbreak (2013) were retrospectively analyzed using WGS and compared with their conventional investigations. Corresponding results were obtained, with WGS delivering even more information, e.g., on virulence and antimicrobial resistance genotypes. Besides an universal, all-in-one workflow with less hands-on-time (five versus seven actual working days for WGS versus conventional), WGS-based cgMLST-typing demonstrated increased resolution. This enabled accurate cluster definition which remained unsolved for the 2013 outbreak, partly due to scarce epidemiological linking with the suspect source. Moreover, it allowed detecting two and one earlier circulating STEC O157:H7 (*stx1*+, *stx2*+, *eae*+) and STEC O157:H7 (*stx2*+, *eae*+) strains as closely related to the 2012 and 2013 outbreaks, respectively, which might have further directed epidemiological investigation initially. Although some bottlenecks concerning centralized data-sharing, sampling strategies, and perceived costs should be considered, we delivered a proof-of-concept that even in smaller countries, WGS offers benefits for outbreak investigation, if sufficient budget is available to ensure

its implementation in surveillance. Indeed, applying a database with background isolates is critical in interpreting isolate relationships to outbreaks, and leveraging the true benefit of WGS in outbreak investigation and/or prevention.

Keywords: whole genome sequencing, WGS, foodborne outbreak investigation, Shiga Toxin-producing *Escherichia coli*, STEC, food safety, surveillance

CONTENTS

Supplementary Tables 3

1. Table S1: Characteristics of all background isolates determined with conventional methods and WGS 3

2. Table S2: WGS-based serotype determination 7

3. Table S3: WGS-based virulence allele typing 1

4. Table S4: NCBI SRA accession numbers of WGS data of all isolates 3

Supplementary Figures 5

1. Figure S1: Conventional typing results of the Limbug 2012 outbreak 5

Supplementary Methods 6

1. Conventional ISO workflows for STEC detection, isolation and identification from food samples, as performed during the initial outbreak investigation 6

References 7

Supplementary Tables

1. Table S1: Characteristics of all background isolates determined with conventional methods and WGS

Table S1. The characteristics of all background isolates determined with conventional methods and WGS.

Strain ref.	Isolation date (dd/mm/yyyy)	Origin	Conventional methods								WGS																												
			Serotype	Virulence genes						AMR	Serotype	Virulence genes																			AMR genes								
				stx1	*stx2*	*eae*	*ehxA*	*aggR*	*aaiC*			*stx1*	*stx2*	*eae*	*ehxA*	*aggR*	*aaiC*	*katP*	*toxB*	*astA*	*cdtB*	*celB*	*esp*	*gad*	*nle*	*tir*	*iss*	*etpD*	*iha*	*tccP*	*bla*$_{TEM-1B}$	*catA1*	*str*	*sul*	*tet*	*aadA1*	*dfrA*	*ermB*	*mdfA*
**EH1819**	28/7/2009	Human	O157:H7	-	+	+	+	-	-	S	O157:H7	-	c	+	+	-	-	+	+	+	-	-	A B F J P	+	A B C	+	+	+	+	+	-	-	-	-	-	-	-	-	+
**EH1829**	11/8/2009	Human	O157:H7	+	+	+	+	-	-	AMP, CHL, STR, SUL, TET	O157:H7	a	c	+	+	-	-	+	+	+	-	-	A B F J P	+	A B C	+	+	+	+	+	+	+	A B	2	A	-	-	-	+
**EH2012**	18/1/2011	Human	O157:H7	-	a	+	+	-	-	S	O157:H7	-	a	+	+	-	-	+	+	+	-	-	A B F J P	+	A B C	+	+	+	+	+	-	-	-	-	-	-	-	-	+
**EH2130**	13/3/2012	Human	O157:H7	a	a	+	+	-	-	STR, SXT, SUL, TET	O157:H7	a	a	+	+	-	-	+	+	+	-	-	A B F J P	+	A B C	+	+	+	+	+	-	-	-	1	A	+	1	+	+
**EH2131**	13/3/2012	Human	O157:H7	a	a	+	+	-	-	STR, SXT, SUL,TET	O157:H7	a	a	+	+	-	-	+	+	+	-	-	A B F J P	+	A B C	+	+	+	+	+	-	-	-	1	A	+	1	+	+
**EH2216**	12/9/2012	Human	O157:H7	-	a	+	+	-	-	S	O157:H7	-	a	+	+	-	-	+	+	+	-	-	A B F J P	+	A B C	+	+	+	+	+	-	-	-	-	-	-	-	-	+
**EH2217**	12/9/2012	Human	O157:H7	-	a	+	+	-	-	S	O157:H7	-	a	+	+	-	-	+	+	+	-	-	A B F J P	+	A B C	+	+	+	+	+	-	-	-	-	-	-	-	-	+
**EH2220**	20/9/2012	Human	O157:H7	-	a	+	+	-	-	S	O157:H7	-	a	+	+	-	-	+	+	+	-	-	A B F J P	+	A B C	+	+	+	+	+	-	-	-	-	-	-	-	-	+

**TIAC1218**	14/8/2008	Pork meat	O157:H7	-	+	+	NA	NA	NA	NA	O157:H7	-	a	+	+	-	-	+	+	+	-	-	A B F J P	+	A B C	+	+	+	+	+	-	-	A B	2	B	-	-	-	+
**TIAC1354**	7/2/2006	Bovine carcass	O157:H7	-	+	+	NA	NA	NA	NA	O157:H7	-	c	+	+	-	-	+	+	+	-	-	A B F J P	+	A B C	+	+	+	+	+	-	-	-	-	-	-	-	-	+
**TIAC1356**	16/2/2006	Bovine carcass	O157:H7	-	+	+	NA	NA	NA	NA	O157:H7	-	c	+	+	-	-	+	+	+	-	-	A B F J P	+	A B C	+	+	+	+	+	-	-	A B	2	B	-	-	-	+
**TIAC1369**	24/5/2006	Bovine carcass	O157:H7	-	+	+	NA	NA	NA	NA	O157:H7	-	c	+	+	-	-	+	+	+	-	-	A B F J P	+	A B C	+	+	+	+	+	-	-	-	-	-	-	-	-	+
**TIAC1372**	21/6/2006	Bovine carcass	O157:H7	-	+	+	NA	NA	NA	NA	O157:H7	-	a	+	+	-	-	+	+	+	-	-	A B F J P	+	A B C	+	+	+	+	+	-	-	-	-	-	-	-	-	+
**TIAC1382**	27/7/2006	Bovine carcass	O157:H7	-	+	+	NA	NA	NA	NA	O157:H7	-	c	+	+	-	-	+	+	+	-	-	A B F J P	+	A B C	+	+	+	+	+	-	-	-	-	-	-	-	-	+
**TIAC1398**	11/9/2006	Bovine carcass	O157:H7	-	+	+	NA	NA	NA	NA	O157:H7	-	b	+	+	-	-	+	+	+	-	-	A B F J P	+	A B C	+	+	+	+	+	-	-	-	-	-	-	-	-	+
**TIAC1399**	11/9/2006	Bovine carcass	O157:H7	-	+	+	NA	NA	NA	NA	O157:H7	-	b	+	+	-	-	+	+	+	-	-	A B F J P	+	A B C	+	+	+	+	+	-	-	-	-	-	-	-	-	+
**TIAC1400**	11/9/2006	Bovine carcass	O157:H7	-	+	+	NA	NA	NA	NA	O157:H7	-	b	+	+	-	-	+	+	+	-	-	A B F J P	+	A B C	+	+	+	+	+	-	-	-	-	-	-	-	-	+
**TIAC1402**	25/9/2006	Bovine carcass	O157:H7	-	+	+	NA	NA	NA	NA	O157:H7	-	c	+	+	-	-	+	+	+	-	-	A B F J P	+	A B C	+	+	+	+	+	-	-	-	-	-	-	-	-	+
**TIAC1408**	11/10/2006	Cheese	O157:H7	-	+	+	NA	NA	NA	NA	O157:H7	-	c	+	+	-	-	+	+	+	-	-	A B F J P	+	A B C	+	+	+	+	+	-	-	-	-	-	-	-	-	+
**TIAC1411**	16/11/2006	Bovine carcass	O157:H7	-	+	+	NA	NA	NA	NA	O157:H7	-	c	+	+	-	-	+	+	+	-	-	A B F J P	+	B C	+	+	+	+	+	-	-	-	-	-	-	-	-	+
**TIAC1419**	18/1/2005	Meat	O157:H7	-	+	+	NA	NA	NA	NA	O157:H7	-	-	+	+	-	-	+	+	+	-	-	A B F J P	+	A B C	+	+	+	+	+	-	-	-	-	-	-	-	-	+
**TIAC1420**	28/1/2005	Meat	O157:H7	-	+	+	NA	NA	NA	NA	O157:H7	-	c	+	+	-	-	+	+	+	-	-	A B F J P	+	A B C	+	+	+	+	+	-	-	-	-	-	-	-	-	+
**TIAC1426**	10/3/2005	Minced meat	O157:H7	-	+	+	NA	NA	NA	NA	O157:H7	-	a	+	+	-	-	+	+	+	-	-	A B F J P	+	A B C	+	+	+	+	+	-	-	-	-	-	-	-	-	+

**TIAC1428**	16/3/2005	Bovine carcass	O157:H7	-	+	+	NA	NA	NA	NA	O157:H7	-	c	+	+	-	-	+	+	+	-	-	A B F J P	+	A B C	+	+	+	+	+	-	-	-	-	-	-	-	-	+
**TIAC1435**	22/4/2005	Bovine carcass	O157:H7	-	+	+	NA	NA	NA	NA	O157:H7	-	a	+	+	-	-	+	+	+	-	-	A B F J P	+	A B C	+	+	+	+	+	-	-	-	-	-	-	-	-	+
**TIAC1440**	20/5/2005	Bovine carcass	O157:H7	-	+	+	NA	NA	NA	NA	O157:H7	-	a	+	+	-	-	+	+	+	-	-	A B F J P	+	A B C	+	+	+	+	+	-	-	-	-	-	-	-	-	+
**TIAC1442**	2/6/2005	Meat	O157:H7	+	+	+	NA	NA	NA	NA	O157:H7	a	c	+	+	-	-	+	+	+	-	-	A B F J P	+	A B C	+	+	+	+	+	-	-	A B	2	-	-	-	-	+
**TIAC1448**	5/7/2005	Bovine carcass	O157:H7	+	+	+	NA	NA	NA	NA	O157:H7	a	c	+	+	-	-	+	+	+	-	-	A B F J P	+	A B C	+	+	+	+	+	-	-	-	-	-	-	-	-	+
**TIAC1449**	5/7/2005	Bovine carcass	O157:H7	-	+	+	NA	NA	NA	NA	O157:H7	-	c	+	+	-	-	+	+	+	+	-	A B F J P	+	A B C	+	+	+	+	+	-	-	-	-	-	-	-	-	+
**TIAC1454**	19/7/2005	Bovine carcass	O157:H7	-	+	+	NA	NA	NA	NA	O157:H7	-	c	+	+	-	-	+	+	+	-	-	A B F J P	+	A B C	+	+	+	+	+	+	-	A B	2	-	-	8	-	+
**TIAC1460**	28/8/2005	Bovine carcass	O157:H7	-	+	+	NA	NA	NA	NA	O157:H7	-	c	+	+	-	-	+	+	+	-	-	A B F J P	+	A B C	+	+	+	+	+	-	-	-	-	-	-	-	-	+
**TIAC1463**	2/9/2005	Bovine carcass	O157:H7	-	+	+	NA	NA	NA	NA	O157:H7	-	c	+	+	-	-	+	+	+	-	-	A B F J P	+	A B C	+	+	+	+	+	-	-	-	-	-	-	-	-	+
**TIAC1464**	9/9/2005	Bovine carcass	O157:H7	-	+	+	NA	NA	NA	NA	O157:H7	-	a	+	+	-	-	+	+	+	-	-	A B F J P	+	A B C	+	+	+	+	+	-	-	-	-	-	-	-	-	+
**TIAC1472**	20/9/2005	Bovine carcass	O157:H7	-	+	+	NA	NA	NA	NA	O157:H7	-	c	+	+	-	-	+	+	+	-	-	A B F J P	+	A B C	+	+	+	+	+	-	-	-	-	-	-	-	-	+
**TIAC1475**	27/9/2005	Bovine carcass	O157:H7	-	+	+	NA	NA	NA	NA	O157:H7	-	c	+	+	-	-	+	+	+	-	-	A B F J P	+	A B C	+	+	+	+	+	-	-	-	-	-	-	-	-	+
**TIAC1477**	20/10/2005	Bovine carcass	O157:H7	-	+	+	NA	NA	NA	NA	O157:H7	-	c	+	+	-	-	-	+	+	+	-	A B F J P	+	A B C	+	+	+	+	+	-	-	-	-	-	-	-	-	+
**TIAC1478**	25/10/2005	Bovine carcass	O157:H7	-	+	+	NA	NA	NA	NA	O157:H7	-	c	+	+	-	-	+	+	+	-	+	A B F J P	+	A B C	+	+	+	+	+	-	-	-	-	-	-	-	-	+
**TIAC1479**	25/10/2005	Bovine carcass	O157:H7	-	+	+	NA	NA	NA	NA	O157:H7	-	c	+	+	-	-	+	+	+	-	+	A B F J P	+	A B C	+	+	+	+	+	-	-	-	-	-	-	-	-	+

**TIAC1653**	16/5/2007	Bovine carcass	O157:H7	+	+	+	NA	NA	NA	NA	O157:H7	a	c	+	+	-	-	+	+	+	-	-	A B F J P	+	A B C	+	+	+	+	+	-	-	A B	2	-	-	-	-	+
**TIAC1664**	17/7/2007	Meat	O157:H7	-	+	+	NA	NA	NA	NA	O157:H7	-	c	+	+	-	-	+	+	+	-	-	A B F J P	+	A B C	+	+	+	+	+	-	-	-	-	-	-	-	-	+
**TIAC1888**	9/6/2011	Bovine carcass	O157:H7	+	+	+	NA	NA	NA	NA	O157:H7	a	c	+	+	-	-	+	+	+	-	-	A B F J P	+	A B C	+	+	+	+	+	-	-	-	-	-	-	-	-	+

All background isolates originating from food matrices or swabs are indicated with a TIAC-number, and isolates originating from human stools are indicated with a EH-number. The genotypic characteristics of all isolates with the conventional method (serotyping and virulence gene detection) and WGS (serotyping, virulence and AMR genotyping; determined with read mapping-based gene detection (SRST2) using the SerotypeFinder, VirulenceFinder, and ResFinder databases, respectively) are visually indicated in green (+; present), red (-; absent) and grey (NA ; not tested according to the ISO/TS12126:2012, and ISO 16654:2001). The detected subtypes of a gene (e.g. *stx*, *esp*, *nle*, etc.) are specified. Food isolates are not routinely phenotypically tested for AMR (indicated as not applicable; NA). Phenotypic AMR susceptibility is indicated as S, whereas resistance is indicated with the abbreviation of the respective antibiotic (Ampicillin (AMP), Chloramphenicol (CHL), Streptomycin (STR), Sulfonamides (SUL), Tetracycline (TET)). Isolates whose reference number is shown in red had a similar (EH2216, EH2217, and EH2220) or identical (EH2012, EH2130, and EH2131) IS629 fingerprints compared to those of the Limburg 2012 (EH2130 and EH2131) or Flanders 2013 (EH2012, EH2216, EH2217, and EH2220) outbreak isolates.

foods

MDPI

2. *Table S2: WGS-based serotype determination*

Table S2. WGS-based serotype determination

Isolate reference	Outbreak	O-determining allele		O-type	H-determining allele	H-type
		wzx	*wzy*		*fliC*	
**EH2165**	2012	199	200	O157	15	H7
**EH2169**	2012	199	201	O157	15	H7
**EH2170**	2012	201	201	O157	15	H7
**EH2171**	2012	199	200	O157	15	H7
**EH2174**	2012	201	200	O157	15	H7
**EH2176**	2012	199	200	O157	15	H7
**EH2177**	2012	199	200	O157	15	H7
**EH2178**	2012	199	200	O157	15	H7
**EH2179**	2012	199	200	O157	15	H7
**EH2180**	2012	199	200	O157	15	H7
**EH2181**	2012	201	201	O157	15	H7
**EH2182**	2012	199	200	O157	15	H7
**EH2183**	2012	201	200	O157	15	H7
**EH2189**	2012	201	201	O157	15	H7
**EH2192**	2012	199	200	O157	15	H7
**EH2193**	2012	199	200	O157	15	H7
**EH2194**	2012	199	200	O157	15	H7
**TIAC1150**	2012	199	201	O157	15	H7
**TIAC1151**	2012	201	201	O157	15	H7
**TIAC1152**	2012	199	201	O157	15	H7
**TIAC1153**	2012	199	200	O157	15	H7
**EH2264**	2013	201	200	O157	15	H7
**EH2268**	2013	201	200	O157	15	H7
**EH2272**	2013	199	200	O157	15	H7
**EH2274**	2013	201	201	O157	15	H7
**EH2275**	2013	201	201	O157	15	H7
**EH2277**	2013	199	200	O157	15	H7
**EH2278**	2013	199	200	O157	15	H7
**EH2279**	2013	199	200	O157	15	H7
**EH2280**	2013	199	200	O157	15	H7
**EH2281**	2013	199	201	O157	15	H7
**EH2285**	2013	199	200	O157	15	H7
**EH2295**	2013	199	201	O157	15	H7
**EH2297**	2013	201	201	O157	15	H7
**EH2299**	2013	201	200	O157	15	H7
**EH2308**	2013	199	200	O157	15	H7
**TIAC1903**	2013	201	200	O157	15	H7

Serotyping results per isolate of both outbreaks (O), i.e. Limburg 2012 and Flanders 2013, are indicated through the detected O- and H- serotype determining alleles. Results were obtained from WGS data analysis using SRST2 and the SerotypeFinder database. Presence of the respective gene allele is indicated with "+" in a dark green box in case the reference allele was detected with 100.00% coverage and 0.00% sequence divergence, "+" in a light green box in case the reference allele was detected with 100.00% coverage but < 10.00% sequence divergence, or "+" in a grey box in case the reference allele was detected with > 60.00% coverage and < 10.00% sequence divergence. When the reference allele was not detected within these criteria, a "-" in a red box is shown. The SerotypeFinder database determined the O- and H-types from the detected serotyping alleles, based on decision rules [1].

foods MDPI

3. Table S3: WGS-based virulence allele typing

Table S3. WGS-based virulence allele typing results

Isolate reference	Outbreak	*stx1*	*stx2*	*ehxA*	*eae*	*nleA*	*nleB*	*nleC*	*toxB*	*astA*	*etpD*	*katP*	*iha*	*espA*	*espP*	*espB*	*espF*	*espJ*	*tccP*	*iss*	*gad*	*tir*
**EH2165**	2012	7	31	3	42	8	13	4	-	-	1	1	14	19	4	6	-	2	-	-	-	26
**EH2169**	2012	7	31	3	42	8	13	4	-	-	1	1	14	19	4	6	-	2	-	-	-	26
**EH2170**	2012	7	31	3	42	8	13	4	-	-	1	1	14	19	4	6	10	2	-	-	-	26
**EH2171**	2012	7	31	3	42	8	13	4	-	-	1	1	14	19	4	6	-	2	-	-	-	26
**EH2174**	2012	7	31	3	42	8	13	4	-	-	1	1	14	19	4	6	10	2	-	-	-	26
**EH2176**	2012	7	31	3	42	8	13	4	-	-	1	1	14	19	4	6	-	2	-	-	-	26
**EH2177**	2012	7	31	3	42	8	13	4	-	-	1	1	14	19	4	6	-	2	-	-	-	26
**EH2178**	2012	7	31	3	42	8	13	4	-	-	1	1	14	19	4	6	-	2	-	-	-	26
**EH2179**	2012	7	31	3	42	8	13	4	-	-	1	1	14	19	4	6	10	2	-	-	-	26
**EH2180**	2012	7	31	3	42	8	13	4	-	-	1	1	14	19	4	6	10	2	-	-	-	26
**EH2181**	2012	7	31	3	42	8	13	4	-	-	1	1	14	19	4	6	10	2	-	-	-	26
**EH2182**	2012	7	31	3	42	8	13	4	-	-	1	1	14	19	4	6	-	2	-	-	-	26
**EH2183**	2012	7	31	3	42	8	13	4	-	-	1	1	14	19	4	6	10	2	-	-	-	26
**EH2189**	2012	7	31	3	42	-	13	4	-	-	1	1	14	19	4	6	10	2	-	-	-	26
**EH2192**	2012	7	31	3	42	8	13	4	-	-	1	1	14	19	4	6	-	2	-	-	-	26
**EH2193**	2012	7	31	3	42	8	13	4	-	-	1	1	14	19	4	6	-	2	-	-	-	26
**EH2194**	2012	7	31	3	42	8	13	4	-	-	1	1	14	19	4	6	10	2	-	-	-	26
**TIAC1150**	2012	7	31	3	42	8	-	4	-	-	1	1	14	19	4	6	10	2	-	-	6	26
**TIAC1151**	2012	7	31	3	42	8	-	4	-	-	1	1	14	19	4	6	-	2	-	-	6	26
**TIAC1152**	2012	7	31	3	42	8	13	4	-	-	1	1	14	19	4	6	-	2	-	-	-	26

**TIAC1153**	2012	12	52	3	42	8	13	4	-	-	1	1	14	19	-	6	10	2	-	-	-	26
**EH2264**	2013	/	24	3	42	8	13	4	-	-	1	1	14	19	4	6	-	2	-	-	-	-
**EH2268**	2013	/	24	3	42	8	13	4	-	-	1	1	14	19	4	6	10	2	-	-	-	-
**EH2272**	2013	/	24	3	42	8	13	4	-	-	1	1	14	19	4	6	-	2	-	-	-	-
**EH2274**	2013	/	24	3	42	8	-	4	-	-	1	1	14	19	4	6	-	2	-	-	-	-
**EH2275**	2013	/	24	3	42	8	13	4	-	-	1	1	14	19	4	6	-	2	-	-	-	-
**EH2277**	2013	/	24	3	42	8	13	4	-	-	1	1	14	19	4	6	10	2	-	-	-	-
**EH2278**	2013	/	24	3	42	8	13	4	-	-	1	1	14	19	4	6	10	2	-	-	-	-
**EH2279**	2013	/	24	3	42	8	-	4	-	-	1	1	14	19	4	6	10	2	-	-	-	-
**EH2280**	2013	/	24	3	42	8	13	4	-	-	1	1	14	19	4	6	10	2	-	-	-	-
**EH2281**	2013	/	24	3	42	8	13	4	-	-	1	1	14	19	4	6	10	2	-	-	-	-
**EH2285**	2013	/	24	3	42	8	13	4	-	-	1	1	14	19	4	6	10	2	-	-	-	-
**EH2295**	2013	/	24	3	42	8	13	4	-	-	1	1	14	19	4	6	-	2	-	-	-	-
**EH2297**	2013	/	24	3	42	8	13	4	-	-	1	1	14	19	4	6	10	2	-	-	-	-
**EH2299**	2013	/	24	3	42	8	13	4	-	-	1	1	14	19	4	6	10	2	-	-	-	-
**EH2308**	2013	12	24	3	42	8	13	4	-	-	1	1	14	19	4	6	-	2	-	-	-	-
**TIAC1903**	2013	/	24	3	42	8	13	4	-	-	1	1	14	19	4	6	10	2	-	-	-	-

Virulence allele typing results determined with WGS using direct read mapping with SRST2 are indicated for all O157 isolates with similar characteristics, collected during the Limburg 2012 and Flanders 2013 outbreak. The VirulenceFinder database was used. Only alleles for which exact matches with the gene alleles in the VirulenceFinder database were obtained are shown (i.e. 100% query coverage and 0% sequence divergence). When multiple exact matches were obtained, the largest hit is retained. The partial detection of a gene allele (here indicated as "-") are likely new alleles of the gene not present in the database. Detection of *stx1* alleles 7 and 12 encode for the *stx1a* subtype. Detection of *stx2* alleles 24 and 31 encode for the *stx2a* subtype, whereas *stx2* allele 52 encodes for the *stx2c* subtype.

4. Table S4: NCBI SRA accession numbers of WGS data of all isolates

Table S4. NCBI SRA accession numbers of WGS data of all isolates

**Bioproject accession**	**Accession**	**Isolate reference**	**Reference**
PRJNA574887	SRR10201483	TIAC1151	Nouws and Bogaerts et al. 2020 [2]
	SRR10201465	TIAC1152	
	SRR10201452	TIAC1153	
	SRR10201427	EH2165	
	SRR10201416	EH2174	
PRJNA645975	SAMN15521963	EH2169	This study
	SAMN15521964	EH2170	
	SAMN15521965	EH2171	
	SAMN15521966	EH2176	
	SAMN15521967	EH2177	
	SAMN15521968	EH2178	
	SAMN15521969	EH2179	
	SAMN15521970	EH2180	
	SAMN15521971	EH2181	
	SAMN15521972	EH2182	
	SAMN15521973	EH2183	
	SAMN15521974	EH2189	
	SAMN15521975	EH2192	
	SAMN15521976	EH2193	
	SAMN15521977	EH2194	
	SAMN15521978	TIAC1150	
	SAMN15521979	EH2264	
	SAMN15521980	EH2268	
	SAMN15521981	EH2272	
	SAMN15521982	EH2274	
	SAMN15521983	EH2275	
	SAMN15521984	EH2277	
	SAMN15521985	EH2278	
	SAMN15521986	EH2279	
	SAMN15521987	EH2280	
	SAMN15521988	EH2281	
	SAMN15521989	EH2285	
	SAMN15521990	EH2295	
	SAMN15521991	EH2297	
	SAMN15521992	EH2299	

	SAMN15521993	EH2308	
	SAMN15521994	TIAC1903	
	SAMN15521995	EH2012	
	SAMN15521996	EH2130	
	SAMN15521997	EH2131	
	SAMN15521998	EH2216	
	SAMN15521999	EH2217	
	SAMN15522000	EH2220	
PRJNA633966	SRR11816078	EH1819	Bogaerts and Nouws et al. [1]
	SRR11816076	EH1829	
	SRR11816023	TIAC1218	
	SRR11816054	TIAC1354	
	SRR11816049	TIAC1356	
	SRR11816048	TIAC1369	
	SRR11816047	TIAC1372	
	SRR11816046	TIAC1382	
	SRR11816044	TIAC1398	
	SRR11816043	TIAC1399	
	SRR11816042	TIAC1400	
	SRR11816031	TIAC1402	
	SRR11816053	TIAC1408	
	SRR11816030	TIAC1411	
	SRR11816000	TIAC1419	
	SRR11815999	TIAC1420	
	SRR11816041	TIAC1426	
	SRR11816029	TIAC1428	
	SRR11816028	TIAC1435	
	SRR11816027	TIAC1440	
	SRR11815998	TIAC1442	
	SRR11816040	TIAC1448	
	SRR11816026	TIAC1449	
	SRR11816025	TIAC1454	
	SRR11816039	TIAC1460	
	SRR11816024	TIAC1463	
	SRR11816022	TIAC1464	
	SRR11816021	TIAC1472	
	SRR11816020	TIAC1475	
	SRR11816019	TIAC1477	
	SRR11816018	TIAC1478	
	SRR11816017	TIAC1479	

	SRR11815977	TIAC1653	
	SRR11815976	TIAC1664	
	SRR11816002	TIAC1888	

The datasets supporting the conclusions of this study have been deposited in the NCBI SRA under the bioproject accession numbers PRJNA574887, PRJNA645975, and PRJNA633966 (all in-house sequenced data) as listed in the first column. The accession numbers of each isolate is shown.

Supplementary Figures

1. Figure S1: Conventional typing results of the Limbug 2012 outbreak

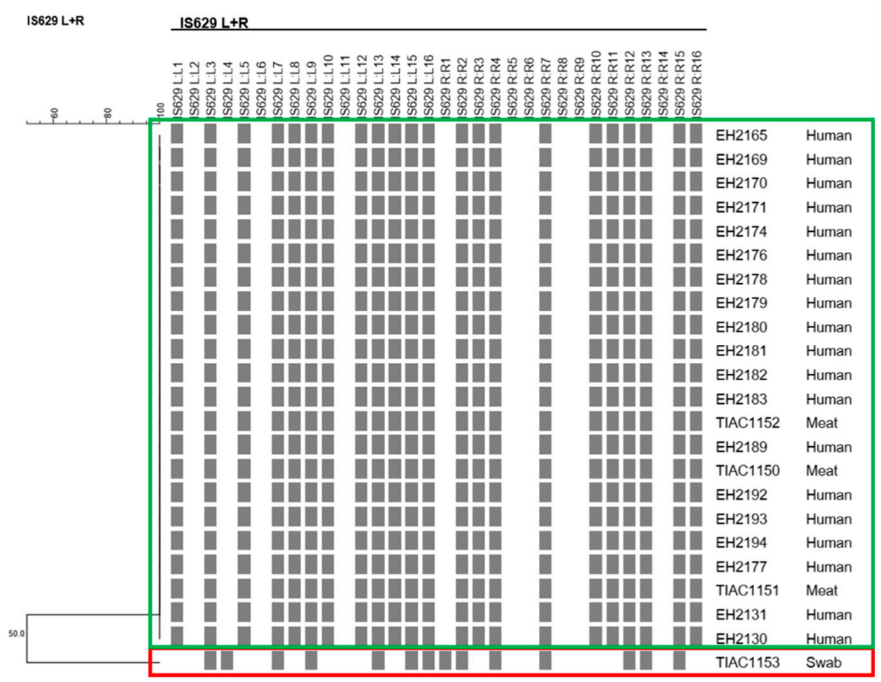

(**a**)

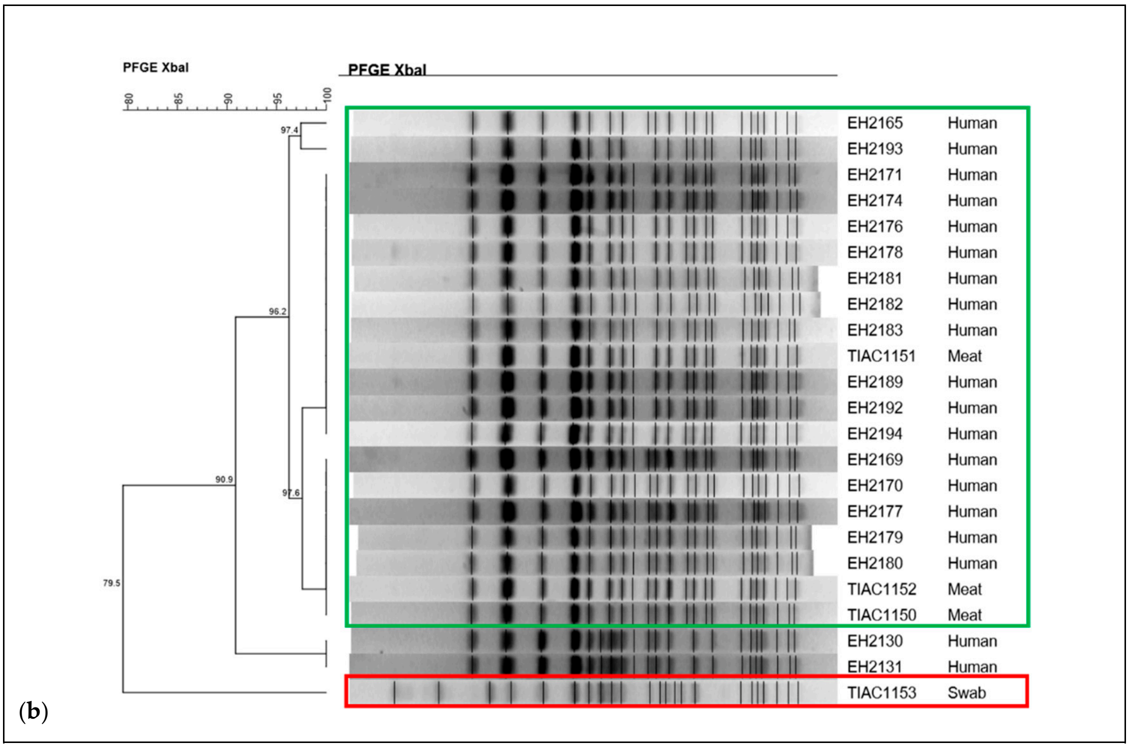

Figure S1. The IS629 fingerprints (**a**) and PFGE patterns (**b**) of all suspected outbreak isolates involved in the Limburg 2012 outbreak are depicted. All isolates that are clustering together are encircled in green. The carcass isolate that had a complete different IS629 fingerprint and PFGE pattern compared to all other outbreak isolates is encircled in red. Two background isolates were identified with identical IS629-types as the isolates collected during the outbreak period.

Supplementary Methods

1. Conventional ISO workflows for STEC detection, isolation and identification from food samples, as performed during the initial outbreak investigation

The initial outbreak investigation was performed according to the conventional methods. The detection, isolation and identification of *E. coli* O157 STEC from food and carcass swab samples was performed at the NRL-STEC according to the ISO/TS 13136:2012 and ISO 16654 workflows, as part of the initial investigation. For the Limburg 2012 outbreak, these procedures were also described by Braeye *et al*. (2014) [3].

Detection of STEC in food was performed according to the ISO/TS 13136:2012 standard. Shortly, 25mg or 25ml of the susceptible matrix or 1 swab was solubilized in 225ml or 90ml of Buffered Peptone Water (BPW; Lab M Limited, Lancashire, UK), respectively, mixed for 1 min in a stomacher bag using a Bagmixer 400 CC (Interscience, Louvain-La-Neuve, Belgium) and incubated at 37±1°C for 18±2h (food samples) or at 41.5±1°C for 6h (carcass swabs). Fifty microliters of the enriched samples was used for 10min of heat lysis DNA extraction at 100°C using the Pall Food Extraction Pack (Pall Corporation, New York, USA) and subsequent qPCR reaction using the GSTEHEC106006 GeneDisc (Pall Corporation, New York, USA) for the detection of major STEC virulence genes (*stx1*, *stx2*, and *eae*) and $rfbE_{O157}$, according to the manufacturer's protocol. When *stx* and *eae* were detected, the Pall Food Extraction Pack and GEHECID106006 GeneDisc were applied for detection of wzx_{O26}, wzx_{O103}, $wbd1_{O111}$, $fliC_{H7}$ and $thp1_{O145}$, according to the manufacturer's protocol.

Isolation of the *E. coli* O157 STEC was performed according to ISO 16654:2001 for detection and isolation of *E. coli* O157 STEC. Shortly, 25g food matrix or 1 swab was mixed in 225ml or 90ml modified Tryptic Soy Broth with 20mg/l Novobiocin (mTSB + n; BioMérieux, Marcy-l'Étoile, France), respectively, using a Bagmixer 400 CC. The samples were incubated at 42±1°C for 6±1h, and again for

18±2h. Separation and concentration of the *E. coli* O157 was executed by immunocapturing, for which 20µl Dynabeads *E. coli* anti-O157 immunomagnetic beads (Invitrogen, California, USA) were added to 1ml enriched sample and processed according to the manufacturer's protocol. Fifty microliters of washed beads were spread on CT-SMAC (BioMérieux, Marcy-l'Étoile, France) and incubated for 18-24h at 37°C for growth. *E. coli* O157 isolates appear as sorbitol negative, colorless colonies. To confirm the presence of these *E. coli* O157, the Oxoid *E. coli* O157 Latex Test (Oxoid Limited, Hampshire, UK) was performed using anti-O157 immunoglobulins, according to the manufacturer's instructions. Confirmation of *E. coli*/*Shigella* was obtained using the MALDI-TOF MS through adding 1µl of HCCA (Bruker, Massachusetts, USA) to a bacterial colony before reading the plate. Confirmation of STEC was performed using qPCR with the Pall Food Extraction Pack and GSTEHEC106006 GeneDisc for *stx1, stx2*, and *eae* detection. The obtained isolates from food and carcass swabs were then transferred to the NRC-STEC for further characterization and study of the relatedness with the human isolates (see main manuscript).

References

1. Bogaerts, B.; Nouws, S.; Verhaegen, B.; Denayer, S.; Braekel, J. Van; Winand, R.; Fu, Q.; Piérard, D.; Marchal, K.; Roosens, N. H. C.; De Keersmaecker, S. C. J.; Vanneste, K. Validation of a Bioinformatics Workflow for Characterization of Shiga Toxin-Producing *Escherichia coli*, Applied to a High-Quality Reference Collection, Demonstrates the Feasibility of Switching to Whole-Genome Sequencing for Routine Pathogen Typing. In Preparation.

2. Nouws, S.; Bogaerts, B.; Verhaegen, B.; Denayer, S.; Piérard, D.; Marchal, K.; Roosens, N. H.; Vanneste, K.; De Keersmaecker, S. C. J. Impact of DNA Extraction on Whole Genome Sequencing Analysis for Characterization and Relatedness of Shiga Toxin-Producing *Escherichia coli* Isolates. *Sci. Rep.* Under Review.

3. Braeye T; Denayer S; De Rauw K; Forier A; Verluyten J; Fourie L; Dierick K; Botteldoorn N; Quoilin S; Cosse P; Noyen J; Pierard D. Lessons Learned from a Textbook Outbreak: EHEC-O157. *Arch. Public Heal.* **2014**, *72* (1), 44.

© 2020 by the authors. Submitted for possible open access publication under the terms and conditions of the Creative Commons Attribution (CC BY) license (http://creativecommons.org/licenses/by/4.0/).
